# Combined effects of human pressures on Europe’s marine ecosystems

**DOI:** 10.1007/s13280-020-01482-x

**Published:** 2021-01-28

**Authors:** Samuli Korpinen, Leena Laamanen, Lena Bergström, Marco Nurmi, Jesper H. Andersen, Juuso Haapaniemi, E. Therese Harvey, Ciaran J. Murray, Monika Peterlin, Emilie Kallenbach, Katja Klančnik, Ulf Stein, Leonardo Tunesi, David Vaughan, Johnny Reker

**Affiliations:** 1 Marine Research Centre, Finnish Environment Institute (SYKE), Helsinki, Finland; 2 Department of Aquatic Resources, Swedish University of Agricultural Sciences, Uppsala, Sweden; 3 NIVA Denmark Water Research, Copenhagen, Denmark; 4 HELCOM Secretariat, Helsinki, Finland; 5 European Environment Agency (EEA), Copenhagen, Denmark; 6 Institute for Water of the Republic of Slovenia (IZVRS), Ljubljana, Slovenia; 7 EcoLogic, Berlin, Germany; 8 Italian National Institute for Environmental Protection and Research (ISPRA), Rome, Italy; 9 Joint Nature Conservation Committee (JNCC), Peterborough, UK

**Keywords:** Anthropogenic pressures, Cumulative effect assessments, Europe's seas, Human activities, Marine assessment, Marine management

## Abstract

Marine ecosystems are under high demand for human use, giving concerns about how pressures from human activities may affect their structure, function, and status. In Europe, recent developments in mapping of marine habitats and human activities now enable a coherent spatial evaluation of potential combined effects of human activities. Results indicate that combined effects from multiple human pressures are spread to 96% of the European marine area, and more specifically that combined effects from physical disturbance are spread to 86% of the coastal area and 46% of the shelf area. We compare our approach with corresponding assessments at other spatial scales and validate our results with European-scale status assessments for coastal waters. Uncertainties and development points are identified. Still, the results suggest that Europe’s seas are widely disturbed, indicating potential discrepancy between ambitions for Blue Growth and the objective of achieving good environmental status within the Marine Strategy Framework Directive.

**Supplementary information:**

The online version of this article (10.1007/s13280-020-01482-x) contains supplementary material, which is available to authorized users.

## Introduction

Europe’s seas support economic prosperity worth billions, and the European Union’s (EU) Blue Growth strategy aims for sustainable development of ‘blue economy’ in established and emerging sectors (European Commission 2020). The Marine Strategy Framework Directive (MSFD, European Commission 2008) is the EU instrument to ensure that the use of seas is on sustainable level and that the marine environment achieves good environmental status (GES) by 2020 or 2024. However, assessments by the Member States (https://water.europa.eu/marine/data-maps-and-tools/msfd-reporting-information-products/msfd-reporting-data-explorer/msfd-start) and coordinated assessments carried out by Regional Sea Conventions (OSPAR 2017; UNEP-MAP 2017; HELCOM 2018a) have documented that GES has not yet been achieved. The two policies—Blue Growth and MSFD—clearly conflict because of structural challenges to meet the two goals simultaneously (Alexander et al. 2015). Elliott et al. (2020a) proposed that this conflict can be alleviated via an integrated framework which merges the natural and human aspects. A part of this system is in understanding of spatial cumulative effects from human activities. Following this, we have estimated the human impacts to seas from the pressure point of view; how widely do human activities and anthropogenic pressures potentially affect Europe’s seas?

Spatially referenced cumulative effect assessments (CEA) map and assess the distribution of key pressures and their potential combined effects over assessment areas (e.g., Halpern et al. 2015). A specific CEA may evaluate how several human activities or pressures can act together on the same environment or visualize the combined effect when one pressure occurs simultaneously in many places over a larger area, hence providing guidance to targeted management actions (Stelzenmüller et al. 2018). CEAs have been carried out for several marine areas and globally (reviewed by Korpinen and Andersen 2016) and methods evolve continuously with regard to assessment approaches and data quality aspects (e.g., Stelzenmüller et al. 2015; Stock and Micheli 2016; Jones et al. 2018; Hodgson et al. 2019). Despite advances in application and computation, a major limiting factor in spatially referenced CEA is still the availability and accuracy of data (Stelzenmüller et al. 2015). Further, data coherence becomes a central issue when targeting wider geographical scales, where data from several different sources need to be combined.

This study presents the results of collating spatial data on human activities and pressures from several sources, at the scale of European marine area, to develop a coherent spatial assessment. We calculated a CEA index across the European marine area to assess the relative extents and magnitudes of pressures and their potential impacts at this scale. This study presents the first combined marine assessment of multiple pressures and their potential effects at this level and detail. By this, (1) we aim to show that spatial pressure-based approaches can be useful tools for marine managers, and that (2) they can complement status monitoring in wide marine areas, and (3) we also suggest that the combined effects in Europe’s seas are currently too extensive to reach GES.

## Method overview

Cumulative effects of human activities on the ecosystem can be evaluated in different ways. Methods vary depending on research or assessment questions, assessed scales, and differences in data availability. Hence, relating different studies to each other is not always straightforward, as further emphasized by differences in applied vocabulary (a useful review is provided by Judd et al. 2015). For instance, the term ‘cumulative impact,’ which is often used, fundamentally refers to the sum of synergistic, antagonistic and additive effects of multiple anthropogenic or natural pressures on the focal environmental aspect (Crain et al. 2008), but it has proven difficult to include all these effects in real assessments (Halpern and Fujita 2013). For spatially referenced assessments, additive effects are the most commonly included (Korpinen and Andersen 2016). At the large scale applied in this study, we include only additive effects and follow the example by Goodsir et al. (2015) to use the term ‘combined-effect assessment’ and the acronym ‘CEA.’

**Fig. 1:**
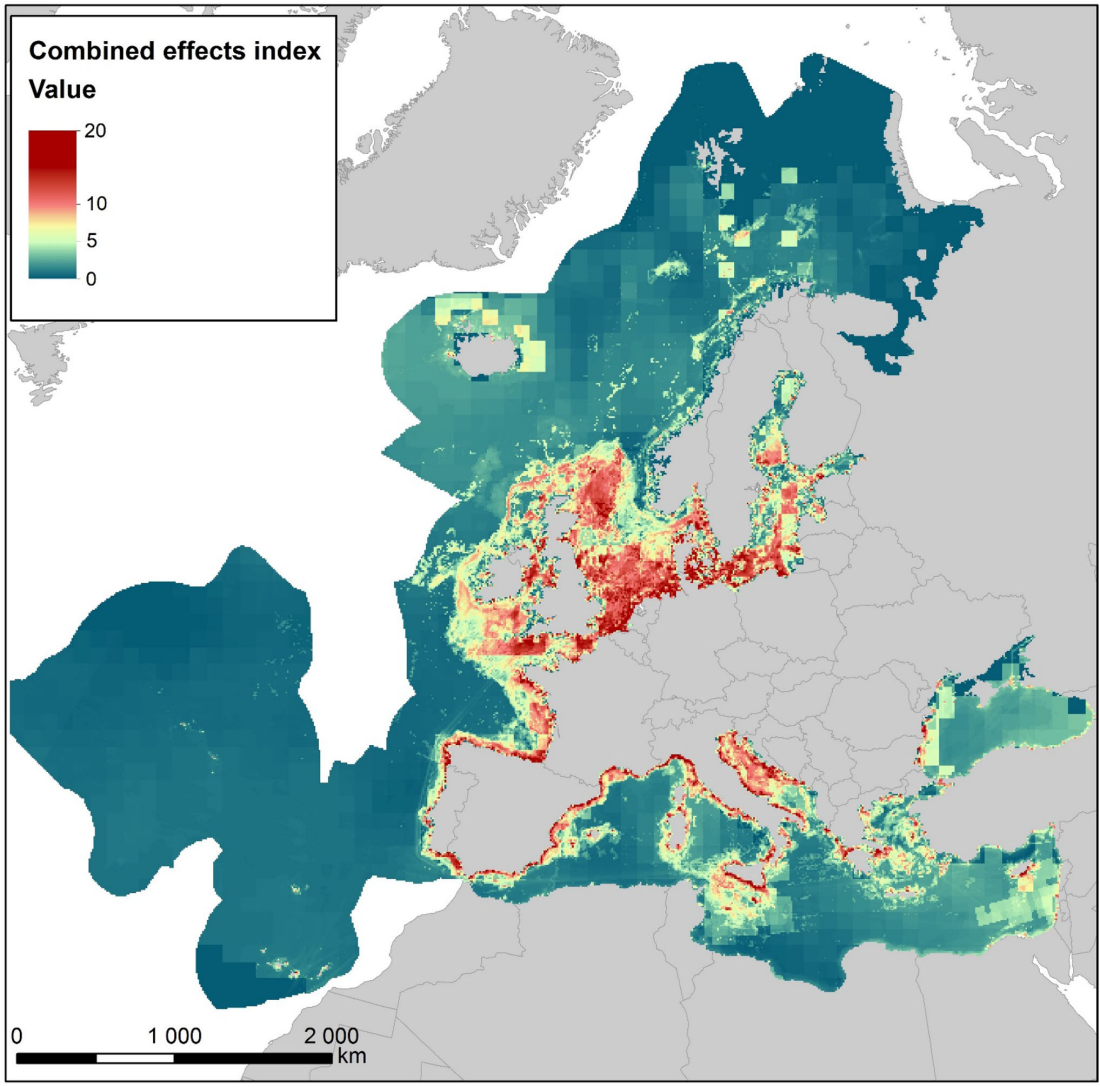
Combined effects of anthropogenic pressures in Europe’s seas. The marine area follows the European Environment Agency’s delineation of the Marine Strategy Framework Directive assessment area

We calculated the combined effects by the method developed by Halpern et al. (2008) and later used several times in Europe (Coll et al. 2012; Korpinen et al. 2012; Micheli et al. 2013; HELCOM 2018a; Bevilacqua et al. 2018; Andersen et al. 2020). The index (*I*) is calculated as follows: ${I}_{\mathrm{CEA}}={\sum }_{i=1}^{n}\frac{1}{m}{\sum }_{j=1}^{m}{P}_{i}\times {E}_{j}\times {\mu }_{i,j}$, where *P*_*i*_ is the log-transformed and normalized value of an anthropogenic pressure (scaled between 0 and 1) in an assessment unit *i*, *E*_*j*_ is the occurrence of an ecosystem component *j* (i.e., habitat, species; scaled between 0 and 1), and *µ*_*i,j*_ is the sensitivity score for *P*_*i*_ in *E*_*j*_ (original values 0–5 scaled between 0 and 1). We calculated the index to 10 km × 10 km grid cells to the entire European marine area (Fig. 1) by using the EcoImpactMapper software (Stock 2016).

Anthropogenic pressures were included based on a common standard, Annex III of the EU MSFD. However, we did not include very local pressures which currently lack relevance at European scale (brine water inputs, water extraction, introductions of genetically modified species, and translocations of native species were excluded), and also spatial data on marine litter do not exist on European scale. In addition to the MSFD list, we also included bycatches from two types of fishing gears and a layer on sea-surface temperature to represent effects of climate change (14 datasets, see Table 1).

**Table 1:** List of anthropogenic pressures covering the Europe’s seas. See also Appendix S1

Introductions of non-indigenous species	Physical disturbance to seabed
Input of microbial pathogens	Changes to hydrological conditions
Disturbance of species due to human presence	Inputs of nutrients
Extraction of species by commercial fishing	Input of hazardous substances (incl. synthetic and non-synthetic)
Bycatch by pelagic towed gears	Input of continuous anthropogenic sound
Bycatch by bottom-touching mobile gears	Input of impulsive anthropogenic sound
Physical loss of seabed	Sea-surface temperature (not part of the EU MSFD)

Spatial data on habitats and species groups were included based on broad habitat types as defined by Emodnet (https://www.emodnet.eu/seabed-habitats) to give a systematic representation by key substrates and bathymetry. Additionally, we included datasets on mobile species (fish, marine mammals, sea birds), relevant seafloor structures (i.e., seamounts), biogenic benthic habitats (e.g., seagrass), and pelagic habitats, which we identified as widely distributed and impacted by several pressures but not well represented by the broad habitat-type data layers (Table 2).

**Table 2:** List of marine habitats and species groups used in the assessment. See also Appendix S1

Broad habitat types (Emodnet)	Mobile species
Infralittoral rock and biogenic reef	Small toothed cetaceans
Infralittoral coarse sediment	Deep diving toothed cetaceans
Infralittoral mixed sediment	Baleen whales
Infralittoral sand	Seals
Infralittoral mud	Turtles
Circalittoral rock and biogenic reef	Breeding birds
Circalittoral coarse sediment	Fish
Circalittoral mixed sediment	*Biogenic benthic habitats*
Circalittoral sand	Saltmarshes
Circalittoral mud	Seagrasses
Offshore circalittoral coarse sediment	Cold-water corals and other coralligenous formations
Offshore circalittoral mixed sediment	
Offshore circalittoral mud	*Relevant seafloor structures*
Offshore circalittoral rock and biogenic reef	Seamounts
Offshore circalittoral sand	
Bathyal rock and biogenic reef (Cold-water corals)	*Pelagic habitats*
Bathyal seabed (all substrates)	Coastal water column habitat
Abyssal seabed (all substrates)	Offshore water column habitat

Data were collected for the entire European marine area for the period 2011–2016 (see map in Fig. 1). As the grid cell size for the data was 10 km × 10 km, pressure extents are overestimations even if considering impact ranges which expand widely beyond the activity or pressure ranges (Elliott et al. 2020b, see also Discussion). Methods to develop all spatial data layers are described in Appendix S1.

Factors representing the sensitivity of each ecosystem component to each pressure, µ_i, j_, were developed following Teck et al. (2010), as described in Appendix S2.

Sensitivity scores, which estimate the relative sensitivity of different species and habitats to the assessed pressures, have previously been developed for global (Micheli et al. 2013; Halpern et al. 2008, 2015) and regional assessments (Korpinen et al. 2012; HELCOM 2018a; Hammar et al. 2020). However, to our knowledge, there are no studies concluding how generally applicable the results from these studies are. For this European study, we initially considered separate sets of sensitivity scores for each of the four marine regions—Baltic Sea, Black Sea, Mediterranean Sea, and Northeast Atlantic Ocean. Scores were developed based on an online survey approaching experts in each region, as carried out under the European Environment Agency’s task for the European Topic Centre for Inland, Coastal, and Marine waters. The survey gathered responses from 45 experts in total from the Black Sea, Mediterranean Sea, and Northeast Atlantic Ocean regions. The Baltic Sea results were derived from a recent survey of similar design by HELCOM (2018b), which was responded to by 81 Baltic experts. The scores obtained from each of the surveys were highly similar and did not statistically differ in 394 cases out of 450 (87%; *α* = 0.05). For the assessment, we therefore applied identical scores for the whole assessment area, using median values for all the four regions (see methods and regional results in Appendix S2).

## Wide extent of combined effects in Europe’s seas

**Fig. 2:**
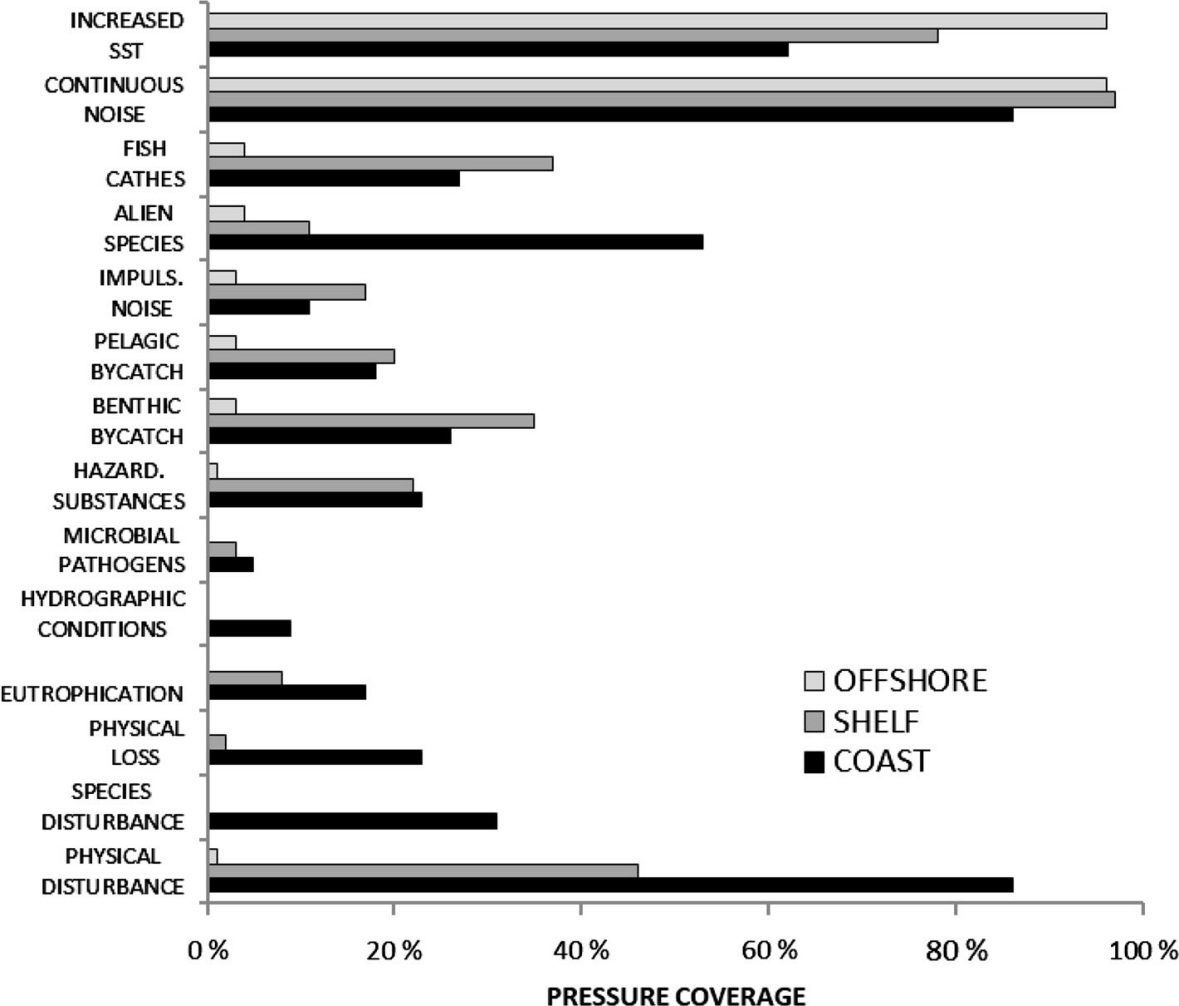
Relative spatial extent of anthropogenic pressures in the coastal area (black), continental shelf (dark gray) area, and beyond shelf (light gray)

Wide areas of Europe’s seas are under influence from human activities and the combined pressures they exert (Fig. 1). According to the results, potential combined effects are relatively highest in the coastal area, slightly lower in shelf areas and decreasing in areas beyond the shelf (Table 3). The analysis reflects that many pressures are more wide-spread on coasts and the continental shelf than in areas beyond the shelf (Fig. 2). For example, ‘species disturbance by human presence’ and ‘hydrographical changes’ occur only in coastal areas, and ‘non-indigenous species’ as well as ‘physical losses’ clearly dominate there. Other pressures, such as ‘physical disturbance,’ ‘input of nutrients,’ ‘input of hazardous substances,’ and the two bycatch pressures are also common in the shelf area. The results indicate that 86% of the coastal grid cells and 46% of the cells in the shelf area were affected by physical disturbance (Fig. 2). Only two pressures were most wide-spread beyond the shelf: ‘increased sea-surface temperature’ and ‘underwater noise.’

**Table 3:** Combined-effects index scores for the coastal area, continental shelf and beyond, respectively. The maximum score is the highest value occurring in any grid cell (10 km × 10 km) within each area, and the mean score is the average for all grid cells in each zone

	Coastal area (0–10 km from coastline)	Continental shelf (more than 10 km offshore, below 1000 m depth)	Beyond continental shelf (deeper than 1000 m)
Maximum score	20.08	17.65	10.00
Mean score	3.80	2.95	0.97

According to the results, the most wide-spread effects at the level of the whole assessed area may be attributed to pressures related to fishing (‘extraction of species,’ ‘bycatch,’ and ‘physical disturbance’), as well as global warming (‘increased sea-surface temperatures’) and shipping (‘underwater noise’) (Fig. 2). Among these, an increase in sea-surface temperature has been clearly documented in European waters with the highest rates of increases in the north (Baltic Sea) (EEA 2017); its role in determining combined effects was ca. 10–15% in coastal waters and 70–95% in offshore waters where other pressures are infrequent or absent. The extent and impacts of fishing have been reported widely across Europe (e.g., Bo et al. 2014; Pusceddu et al. 2014, Eigaard et al. 2017). Regarding underwater noise, there is still insufficient knowledge for European marine waters. Models on underwater noise levels are available for the Baltic Sea (HELCOM 2018a) and Adriatic Sea (Codarin and Picciulin 2015) and are being developed in many parts of the Europe, but the current assessment was based on records of shipping tracks, which may underestimate the extent of this pressure.

Regarding the differences between marine areas, effects of land-based pollution (‘input of nutrients,’ ‘input of hazardous substances,’ ‘input of organic matter,’ ‘input of microbial pathogens’) were clearly indicated in coastal areas and in the semi-enclosed Baltic and Black Seas, which are under strong influence from some of Europe’s largest rivers (Borysova et al. 2005; Artioli et al. 2008, HELCOM 2018c). Pressures related to bottom-trawling fisheries (mainly ‘physical disturbance’) were most evident in the Mediterranean Sea, the Bay of Biscay, the Iberian coast, which are characterized by narrow shelf areas, and the shallow North Sea (Appendix S1).

The maritime sectors contributing most to potential physical disturbance on the seabed were fisheries, related to 55% of the physical disturbance and shipping, related to about one fourth of the physical disturbance. Effects of bottom trawling of the seabed have been documented globally and are connected with significant effects on benthic biodiversity (Hiddink et al. 2017, OSPAR 2017). The effects from shipping on physical disturbance occur on shallow seabed areas and close to the shore, whereas ports and anchoring sites are the main contributors to physical loss of seabed (46% of this pressure’s distribution). Another main contributor to ‘physical loss’ is dredging and dumping (25%), which is linked to the maintenance of shipping lanes and ports, and marine installations (such as wind turbines and oil rigs, 18%).

## What can the CEAs tell us about the state of Europe’s seas?

The state of the environment is formally assessed by biological, chemical or physical indicators which convey information on anthropogenic disturbance and are assessed in relation to threshold values for good status (Zampoukas et al. 2013). While such indicator-based assessments can provide highly accurate results, monitoring and assessment is typically costly and the spatial coverage and representativity is often limited. Extent of human affected area may, however, be a good indication of the state of environment (Katzanidis et al. 2020), but the results cannot easily be applied to assess the environmental status (Halpern and Fujita 2013).

In the EU, Member States assess the state of their coastal waters under the Water Framework Directive (WFD; European Commission 2000; EEA 2018). Ecological status of assessment units (so-called water bodies) is assessed in five classes (high, good, moderate, poor, and bad) on the basis of pre-identified ecological quality elements, representing phytoplankton, macrophytes, benthic invertebrates, fish in transitional waters, and physico-chemical and hydromorphological characteristics.

To compare the outcomes of the two assessments, we related the European CEA results to the status reported by Member States for each quality element in coastal areas (data from https://www.eea.europa.eu/themes/water/dc). The WFD data generally covered the years 2009–2015, though the exact years differed among countries. Using mean CEA scores for each assessed water body, we explored the relationships between the status (WFD) and the level of pressures (CEA) (generalized linear models by the GENMOD procedure in SAS, using a log-link function and assuming a normal distribution). Data were log-transformed where this improved model fit. Model fit was evaluated by the ratio of deviance and degrees of freedom and by minimizing AIC).

**Fig. 3:**
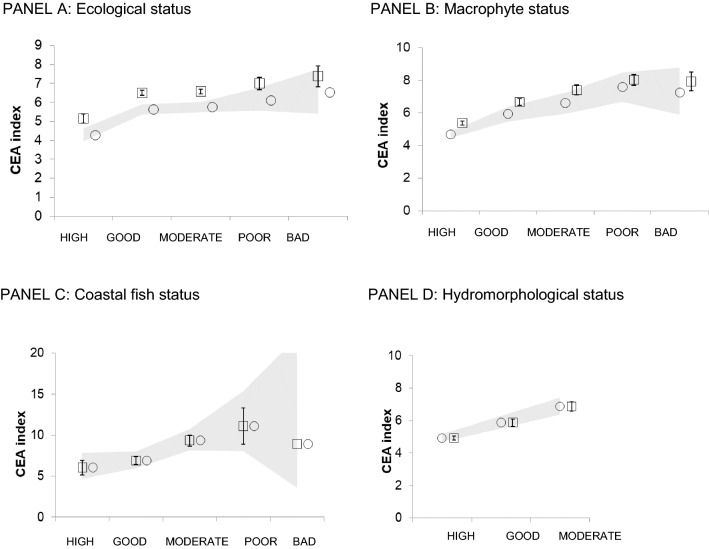
Relationship between coastal water status assessments under the EU Water Framework Directive and the European Combined-Effects Assessment (CEA) index (Fig. 1). **a** ecological status assessment. **b** assessment of macrophyte status. **c** assessment of coastal fish in transitional waters (note that class ‘bad’ has only one observation). **d** hydromorphological status (note that only three status classes were found). Number of waterbodies per assessment are given in text. Key: circles denote predicted values from the model and the green area, the respective 95% CI; squares denote observed values with SE

The results show that a relatively poorer WFD ecological status generally occurs in areas which are associated with high combined effects from anthropogenic pressures according to the CEA (Fig. 3; *N* = 1713, *Χ*^2^ = 49.9, *p* < 0.001). Looking at the different quality elements, this response was seen at European scale for macrophytes (Fig. 3; *N* = 1064, *Χ*^2^ = 126, *p* < 0.001), coastal fish (*N* = 137, *Χ*^2^ = 18, *p* = 0.11), and hydromorphological quality (*N* = 959, *Χ*^2^ = 27, *p* < 0.001). As the hydromorphological quality is based on various pressure indicators in member states, the dependency by the analysis result may be exaggerated. Response by phytoplankton and physico-chemical parameters (nitrogen, phosphorus, and water transparency) was only seen in some marine regions but not at the entire European level (data not shown). The response of benthic invertebrates was bell-shaped, which may indicate that other factors than the assessed human activities are influential at poorer status classes (data not shown).

Interestingly, the comparisons in Fig. 3 suggest that CEA index values above 6 might be indicative of a less than good status classification. If one makes a conservative assumption that a similar CEA score will indicate a less than good state also in other, unassessed water bodies, this would imply that 38% of the grid cells in Europe’s coastal areas fail to reach good status (see color scale in Fig. 1). However, the assessment provides only a first overview. For comparison, a recent assessment of the Southern Celtic Sea showed that 80–90% of the muddy, sandy, coarse, and mixed subtidal seabeds are under high pressure and 86% of the entire seafloor in the Greater North Sea and Celtic seas are disturbed by bottom-touching gears (OSPAR 2017). The OSPAR assessment further indicated that in their entire assessment area, 20% of seagrass meadows, 40% of the seamount area, and 50% of the sea pen and burrowing megafauna habitat are under high disturbance from bottom trawling. In support with our results, integrated assessment study from deep sea sites in northeast Atlantic Ocean suggested that sites further away from the shelf are in better state than the ones closer to coast and the indicator ‘extent of human affected area’ described well the state of these sites (Katzanidis et al. 2020).

## Comparison with CEA assessments at other spatial scales

The results can be compared with those of previous assessments of European relevance, carried out at relatively smaller and larger spatial scales: a national scale (Denmark; Andersen et al. 2020), a regional marine sea (Baltic Sea; HELCOM 2018a), and the global scale (Halpern et al. 2015). In brief, the comparable CEAs were resampled (changing raster cell size), intersected, and normalized from 0 to 1 to analyze the patterns of spatial overlap. All four assessments are based on the same method (Halpern et al. 2008) and, therefore, the differences may mainly rise from the used data. Appendix S3 further describes how the comparison was made.

The comparison demonstrates the dependency of the assessment results on the underlying data. For example, the global CEA did not identify the southern North Sea nor several coastal areas around Europe as highly affected, as was the case with the more data-rich European and Baltic CEAs, and as would be in agreement with status assessments identifying coastal areas as heavily disturbed by several pressures (OSPAR 2017, UNEP-MAP 2017; FAO 2018, HELCOM 2018a). Our study at the European scale, on the other hand, included less data compared to the Baltic Sea and Danish studies (HELCOM 2018a, Andersen et al. 2020), and the relatively smaller scale studies more accurately indicated coastal hotspots such as cities and smaller bays. The national scale assessment (Denmark) included the highest number of data layers and indicated stronger effects in both coastal areas and open sea than in any of the larger-scale CEAs. Together, the comparisons show that both the accuracy of data and the selection of data layers will influence the level to which the results can be interpreted (Halpern and Fujita 2013, see also Stelzenmüller et al. 2015, Stock and Micheli 2016). More specific comparisons of the assessments are given in Appendix S3.

## Sensitivity of European habitats and species to anthropogenic pressures

**Fig. 4:**
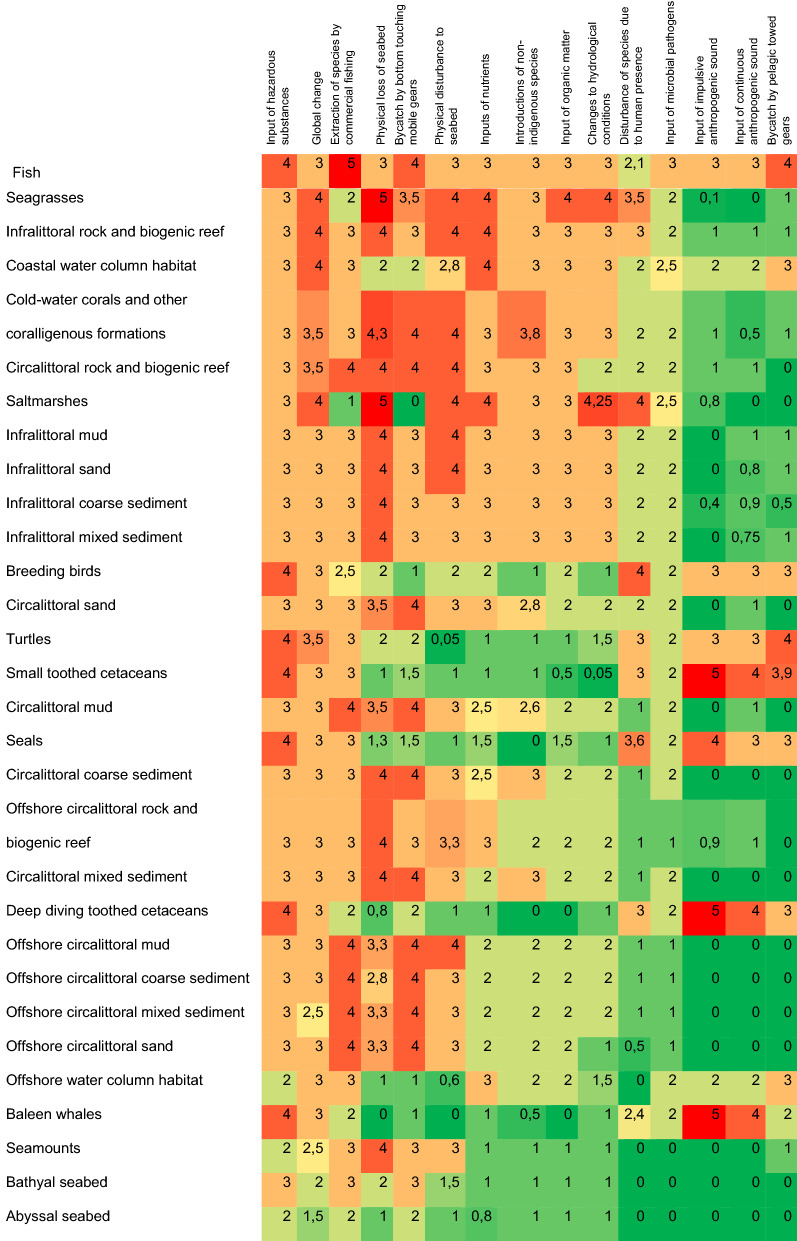
Sensitivity of marine habitats and species against anthropogenic pressures in Europe’s seas. The scores are medians from 0 (not sensitive) to 5 (very sensitive) across all regions and respondents. The color scale represents the scores. Regionally specific scores are given in Appendix S2

The survey among the European marine experts indicated that the Europe’s marine ecosystems are specifically sensitive to extraction of species, increased sea-surface temperature, bycatch of non-target species by fisheries, physical loss of seabed, physical disturbance to seabed, and inputs of hazardous substances and nutrients (Fig. 4).

The sensitivity estimates were collected regionally, and the influence of the region to the sensitivity estimates was tested. Statistically significant differences (*p* < 0.05) were found in 60 of the 450 scores (13%). Differences were found particularly among the sensitivity of infralittoral broad habitats, seagrass, saltmarshes, and seals. In the case of seals, the difference has an obvious reason as the highly threatened monk seal (*Monachus monachus*) is the only Mediterranean seal species and its sensitivity was estimated much higher than the sensitivity of other seal species in the Baltic Sea or NE Atlantic (Black Sea does not have seals at present). We can only guess reasons for the wide range of differences in the infralittoral zone, but this may indicate either differences in experts’ perspective on this zone or biological differences which were not investigated here.

## Discussion

Achieving sustainable use of natural resources and halting the degradation of natural biotic and abiotic systems are major global commitments (Borja et al. 2020; Claudet et al. 2020). In the European Union, which is the focus of the current study, reaching GES of coastal and marine waters is the main objective of the MSFD and vital for Blue Growth and the future development of sea uses. The assessment of how extensively human activities and pressures cover marine areas, and importantly, to identify where pressures are at risk of causing adverse effects is therefore a central issue.

Traditionally, environmental assessments are carried out using state indicators, which are assessed for selected sampling stations with a focus on obtaining reliable estimates of current temporal changes (e.g., Heiskanen et al. 2016; Uusitalo et al. 2016). Modern data portals are offering this data to enable large-scale assessments (Borja et al. 2019). However, the EU MSFD has moved towards more spatial assessments (European Commission 2017), and this increases demands for up-to-date spatial data. We are of the opinion that spatial assessments cannot be achieved using only traditional monitoring by member states but they need support from model-based CEAs. Furthermore, many applications of marine management, including both maritime spatial planning and environmental conservation, require more data-rich approaches and spatial detail. This became also clear when comparing the four CEAs in this study.

Today, CEA applications use data that are often automatically recorded (e.g., automatic identification system “AIS” for shipping, vessel monitoring system “VMS” for fisheries, electronic monitoring system “EMS” for aggregate extraction), stored in permit databases (e.g., marine construction, dredging, dumping, fish catches), or observed from satellites (e.g., sediment plumes, oil spills). This will greatly improve the CEAs which in the past have been limited by data availability, as shown in our brief comparison of CEAs. However, there are still needs for more work in CEA development to include non-linear responses and synergistic and antagonistic effects into the model (Halpern and Fujita 2010, Stock and Micheli 2016). Also the use of region-specific sensitivity values is still an open question, as we have not seen comparisons published before our simple analysis in this study. In our study, we used a simple model but much better data than previously. While this data-rich approach does not advise us of the adverse effects of the pressures on ecosystem status, we have here tentatively explored how the assessment outcomes can be evaluated against state assessments. If a state–pressure relationship is found, the model can potentially be more accurate in space and time than state assessments. In this study, we relied on the widest coherent state assessment in Europe: ecological status of coastal waters. The results indicate a relationship between the pressure and state, which allows some spatial estimations of the level of disturbance in the marine area.

Our results show clearly that Europe’s seas are subject to wide-spread pressures from ongoing human activities, especially in shelf and coastal areas. The CEA results suggest that 38% of the grid cells in coastal waters are in less than good status and, if that threshold is applied to the entire sea area, we estimate that high pressures are spread out to an area of about one fifth of Europe’s seas (19%) (Figs. 1 and 3). Even though this is certainly an overestimation (as the grid cells exaggerate the impact ranges of more local pressures), it gives cause for concern that such a large proportion of Europe’s marine environment is subject to anthropogenic pressures at levels potentially associated with poor ecological status. As spatial resolution of pressure data improves quickly, the CEAs should next use smaller grid cells to better estimate areal estimates of disturbed environment.

We argue that the results indicate a discrepancy between the EU Member State’s ambitions of increased Blue Growth and the objective of achieving good environmental status. While the former strives towards utilizing untapped resources of marine areas and sees potential for economic growth in many traditional and novel sectors, this assessment shows that human activities are not at environmentally sustainable levels and their pressures exert high combined effects on multiple ecosystem components. We further suggest that a significant proportion of these effects may cause disturbed status of the marine environment. Clearly, the EU should first implement stronger management of environmental impacts, spatial planning of their locations (e.g., via the Maritime Spatial Planning Directive, European Commission 2014), as well as concrete actions to conserve marine ecosystem structure and functioning. According to our study and many others, the first step could be to limit physical impacts of bottom trawling, prevent overfishing, and avoid bycatch of marine biota in all the marine regions (Lewison et al. 2014, OSPAR 2017, Avila et al. 2018, FAO 2018, HELCOM 2018a, EEA 2019).

## Supplementary information

Below is the link to the electronic supplementary material.

Supplementary material 1 (PDF 3745 kb)
