# Supplementary material for: Combined effects of human pressures on Europe’s marine ecosystems
Source: Ambio. 2021 Jan 28;50(7):1325–36. doi: 10.1007/s13280-020-01482-x (PMC8116428; doi:10.1007/s13280-020-01482-x)
Supplement: Supplementary file 1 — Supplementary material 1 (PDF 3745 kb) [file 13280_2020_1482_MOESM1_ESM.pdf]

Ambio

Electronic Supplementary Material
This supplementary material has not been peer reviewed

Combined effects of human pressures on Europe's marine ecosystems

Samuli Korpinen*, Leena Laamanen, Lena Bergström, Marco Nurmi, Jesper H. Andersen, Juuso Haapaniemi, E. Therese Harvey, Ciaran J. Murray, Monika Peterlin, Emilie Kallenbach, Katja Klančnik, Ulf Stein, Leonardo Tunesi, David Vaughan, and Johnny Reker

Combined effects of human pressures on Europe’s marine ecosystems

Appendix S1. Development of the spatial data layers

Spatial layers of pressures were developed on the basis of human activities causing them or directly as pressures (e.g. eutrophication). Description of each pressure layer is given in Figure S1.

Spatial layers of ecosystem components were developed for benthic broad habitat types, more specific benthic habitats, two types of pelagic habitats as well as major species groups. Description of each ecosystem component layer is given in Figure S2.

The spatial pressure layers were developed into a grid of 10 km × 10 km cells. Although this may be too coarse for some coastal bays, fjords or archipelagos, it is sufficiently detailed for the offshore area and higher resolution may give over positive impression of the underlying data quality. However, the cell size still overestimates the area of pressure impacts, as many impacts cover only parts of the cell. Similarly, many benthic habitats cover only parts of the cell area. Therefore, the results of this study rather present the geographical spread of combined effects than their areal coverage. Many of the underlying data is in more detailed resolution (see descriptions in Figure S1), but due to coarseness of some spatial layers and limited calculation power, we used the less detailed grid.

Pressure intensity was in some cases indicated by presence or absence of a pressure, but in most cases an intensity score was also given which was estimated either as the intensity of human activity (e.g. shipping or trawling) or amount of a pressure (e.g. pollution). Every pressure layer was scaled between 0 and 1 (or 0/1 in case presence/absence data) and log-transformed, following the method by Halpern et al. 2008, if its distribution had long tails. Spatial extents of pressures were estimated for each type of pressure source (HELCOM 2018) and applied to the layers. As data on physical disturbance and loss did not sufficiently cover the southern Mediterranean (North-Africa), these areas were not included in the interpretation of the pressure distribution in Europe’s seas. Figure S1 further describes the data sources and how each of the pressure layers were produced.

The ecosystem layers were generated to the same area and grid as the pressure layers. The layers of species groups indicate abundance information, whereas habitat layers indicate only presence and absence data (0 or 1). The layers represent the latest known distribution of the features which does not account for the possible decreases in distribution area due to historical pressures. Figure S2 describes the data sources and how the ecosystem layers were produced.

Table S1. Summary description of pressure layers. More detailed descriptions are given in Figure S1.

Pressure layer	Description
Introductions of non-indigenous species	Distribution areas of 76 invasive species. The pressure reflects the number of species.
Input of microbial pathogens	Index of sources or abundance of microbial pathogens based on public beaches, passenger terminals and waste water treatment plants with inadequate treatment.
Disturbance of species due to human presence	Index combining share of urban area in a cell and population density in a cell.
Extraction of species by commercial fishing	Total fish landings intersected with tracks of fishing vessels.
Bycatch by pelagic towed gears	Fishing effort by vessels fishing with pelagic towed gears. As bycatch information is not directly available, the effort shows spatially where these gears are used. Bycatch is potentially higher in areas where effort is higher.
Bycatch by bottom touching mobile gears	As above.
Physical loss of seabed	Activities causing loss of seabed habitat (presence/absence) are summed per cell.
Physical disturbance to seabed	Activities causing disturbance of seabed habitat have either a numeric value (i.e. effort of demersal trawling) and shallow-water shipping) or presence/absence (e.g. dumping sites). The values 0-1 or 0/1 of all activities are summed per grid cell.

Changes to hydrological conditions	All coastal pressures causing hydrographical or hydrological alterations according to the EU Water Framework Directive.
Inputs of nutrients	European Environment Agency's eutrophication assessment integrates nutrient concentrations and effects of eutrophication over the marine area and uses indicator-specific thresholds to depict disturbed areas.
Input of hazardous substances	European Environment Agency's contamination assessment integrates substance concentrations over the marine area and uses substance-specific thresholds to depict disturbed areas.
Input of continuous anthropogenic sound	Maritime traffic is the main source of continuous underwater noise. The traffic density maps are used as a proxy for the pressure.
Input of impulsive anthropogenic sound	International registers for activities causing impulsive noise are used to mark the areas of this pressure and pulse-block days are used as the value.
Sea surface temperature anomaly	Change in mean annual sea surface temperature between 1989-2013 in European seas.

Figure S1. Pressure data and description how the spatial pressure layers were generated.

**Bycatch by bottom touching mobile gears**	
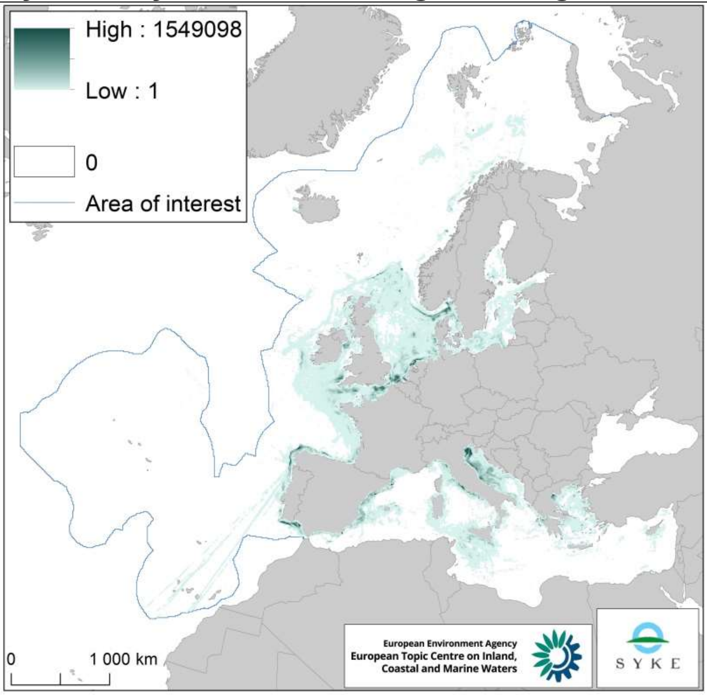	Bycatch by bottom touching mobile gears is estimated on the basis of areas where this type of fishing takes place. It describes areas where bycatch of demersal species is more likely to occur. In the CEA index, overlay with the specific ecosystem component layers (e.g. benthic habitats) will ensure that the bycatch impact to specific ecosystem components can happen only where they occur.<br>Fishing effort (kilowatt per demersal fishing hours per 1 x 1 km grid cell) was created from Automatic Identification System (AIS) based demersal fishing data received from the European Commission's Joint Research Centre - Independent experts of the Scientific, Technical and Economic Committee for Fisheries (JRC STECF). Bottom-touching gears were linked to the AIS id codes from the European fishing vessel register by using the reported primary gear type.<br>This was aggregated (sum) to the EEA 10 x 10 km grid. Data was log-transformed (ln+1).
**Data source:** JRC STECF: https://stecf.jrc.ec.europa.eu/dd/fdi. EU Fleet register: https://webgate.ec.europa.eu/fleet-europa/stat_glimpse_en. The AIS data was available in EU Joint Research Center for their in-house use.	
**Data quality:** Created using AIS data from >=15 meters long fishing vessels. The fishing gear type is an estimate based on the vessel register and not actually reported information.	
**Attribute information:** Sum of kilowatt per fishing hour per 10 x 10 km grid cell.	
**Spatial coverage**: Entire area of interest	
**Spatial resolution**: 1 x 1 km grid aggregated to EEA 10 x 10 km grid.	
**Time period and temporal resolution**: 10.2014 – 9.2015	

Bycatch by pelagic towed gears

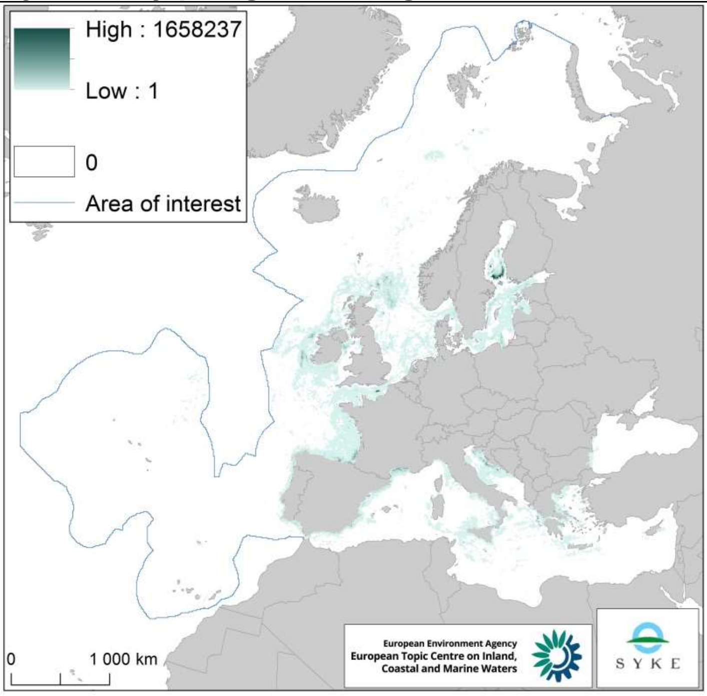

Bycatch by pelagic towed gears is estimated on the basis of areas where this type of fishing takes place. It describes areas where also bycatch of pelagic species is more likely to occur. In the CEA index, overlay with the specific ecosystem component layers (e.g. cetaceans) will ensure that the bycatch impact to specific ecosystem components can happen only where they occur.

Fishing effort (kilowatt per pelagic fishing hours per 1 x 1 km grid cell) was created from Automatic Identification System (AIS) based pelagic fishing data received from the European Commission's Joint Research Centre - Independent experts of the Scientific, Technical and Economic Committee for Fisheries (JRC STECF). Pelagic towed gears were linked to the AIS id codes from the European fishing vessel register by using the reported primary gear type. This was aggregated (sum) to the EEA 10 x 10 km grid. Data was log-transformed (ln+1).

Data source: JRC STECF: https://stecf.jrc.ec.europa.eu/dd/fdi. EU Fleet register: https://webgate.ec.europa.eu/fleet-europa/stat_glimpse_en. The AIS data was available in EU Joint Research Center for their in-house use.

Data quality: Created using AIS data from >=15 meters long fishing vessels. The fishing gear type is an estimate based on the vessel register and not actually reported information.

Attribute information: Sum of kilowatt per fishing hour per 10 x 10 km grid cell.

Spatial coverage: Entire area of interest

Spatial resolution: 1 x 1 km grid aggregated to EEA 10 x 10 km grid.

Time period and temporal resolution: 10.2014 – 9.2015

Extraction of species by commercial fishing

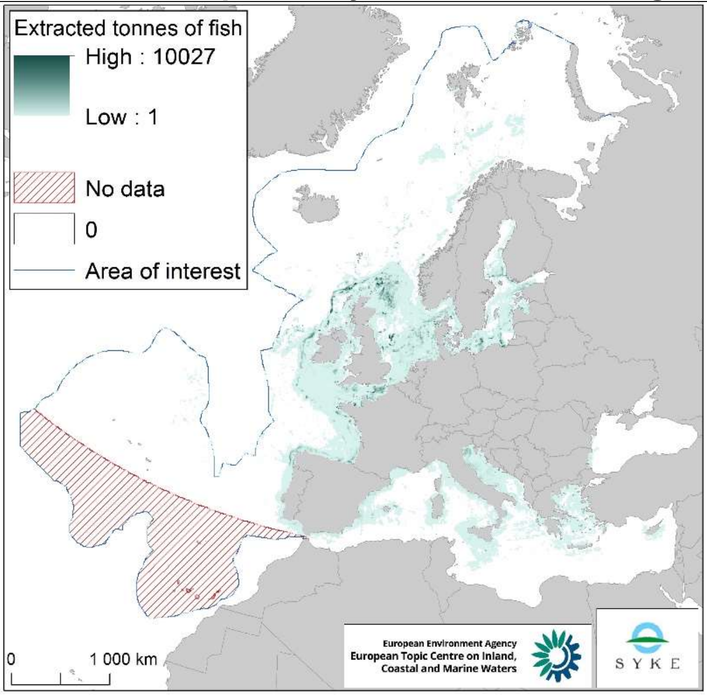

The extraction of species by commercial fishing layer combines demersal and pelagic fishing effort (kw/h aggregated from 1 x 1 km to 10 x 10 km grid) with commercial fishing landings (tonnes per 1° grid cell or, in the case of the Mediterranean sea, tonnes per FAO geographical subareas of differing sizes).

Landing values (in 1° grid cell or FAO subareas) were redistributed to kw/h fishing effort grid cells which had been revalued proportionally within overlapping landing grid cells and subareas.

The resulting layer estimates the amount of extracted fish, in tonnes, per 10 x 10 km grid cell. Data was log-transformed (ln+1).

The southwestern part of the area of interest has no values (no data), as the landings data did not cover this area.

Data source:

See ‘Bycatch from bottom-touching mobile gears’.
**Data quality:** Gaps occurs due to lack of landings data in SW part of the area. As the data layer lacks stationary gears and small-scale fishery, it is an underestimate of the extent of the real pressure.
**Attribute information:** Extracted tonnes of fish per 10 x 10 km grid cell.
**Spatial coverage**: Entire area of interest, except for the most southwestern parts.
**Spatial resolution**: Original data was in raster and polygon format (effort: 1 x 1 km grid, landings: 1° polygon grid, except for the Mediterranean sea which was divided into FAO geographical subareas). Finally the data was converted to the EEA 10 x 10 km grid.
**Time period and temporal resolution**: 01.01.2011–31.12.2016

<table>
<tr><td colspan="2">Disturbance of species due to human presence</td></tr>
<tr><td>
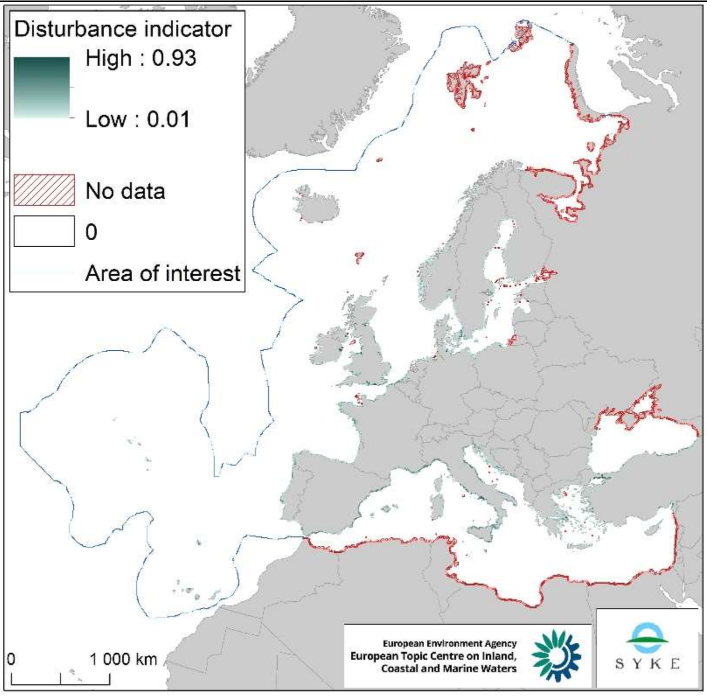

</td><td>The layer presents the amount of disturbance of species due to human presence in EEA 10 x 10 km grid cells. The layer was made by combining coastal urbanisation and population density layers.
The coastal urbanisation layer was derived from the CORINE land cover areas for year 2012 (release v18_5; http://land.copernicus.eu/pan-european/corine-land-cover/clc-2012) by selecting land cover categories 111 Continuous urban fabric, 112 Discontinuous urban fabric, 121 Industrial or commercial units, and 123 Port areas. All these areas were given a 200 m buffer to better reflect the pressure to species. The urbanization value shows the percentage of urbanised coastline per EEA 10 x 10 km grid cell.

The population density layer (http://ec.europa.eu/eurostat/web/population-demography-migration-projections/population-data/database on 24.3.2017 and http://ec.europa.eu/eurostat/web/gisco/geodata/reference-data/administrative-units-statistical-units/nuts on 24.3.2017) shows the population per km^2 in the coastal cells of the EEA 10 x 10 km grid

The pressure layer was made as an index where coastal urbanisation and populations density layers were combined by first log10 transforming and normalising the population density layer, then multiplying the layers with each other.</td></tr>
<tr><td colspan="2">Data source: Corine Land Cover Classification: https://land.copernicus.eu/pan-european/corine-land-cover/clc2018; EUROStat data: https://ec.europa.eu/eurostat/web/products-datasets/product?code=demo_r_d3dens; NUT3 units: https://ec.europa.eu/eurostat/web/gisco/geodata/reference-data/administrative-units-statistical-units.</td></tr>
<tr><td colspan="2">Data quality: Data gaps occur.</td></tr>
<tr><td colspan="2">Attribute information: Species disturbance index calculated from coastal urbanisation and population density using a multiply merge algorithm.</td></tr>
</table>

**Spatial coverage**: Does not include southern and western Mediterranean Sea, northern Black Sea, the northernmost Atlantic Ocean, and some European coastal areas.
**Spatial resolution**: EEA 10 x 10 km grid.
**Time period and temporal resolution**: CORINE 2012, NUTS 2016.

Changes to hydrological conditions

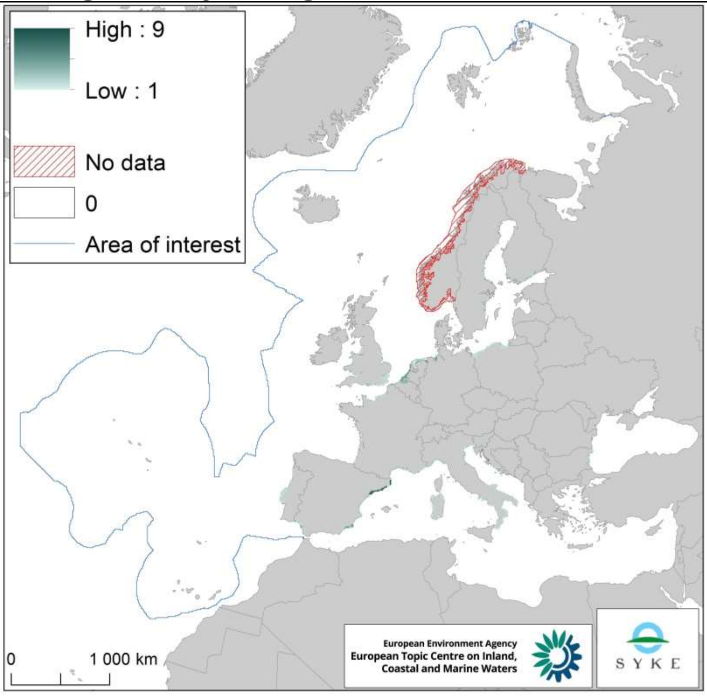

The pressure is defined as a change in hydrology due to anthropogenic structures or activities in coastal waters.

The layer was created using the reported data of pressures under the Water Framework Directive (WFD). The following pressures were selected: (1) physical alteration of channel/ bed/ riparian area/ shore; (2) dams, barriers and locks, and (3) hydrographical alteration.
The WFD data listed the total amount of the selected pressures per water body. This was joined with water body polygon features, downloaded from EEA (https://www.eea.europa.eu/data-and-maps/data/wise-eionet-spatial-2).
The polygon features were then converted to the EEA 10 x 10 km grid, resulting in a raster showing the number of different hydrographical pressures per grid cell.

**Data source:** EEA (Water Framework Directive reporting: https://www.eea.europa.eu/data-and-maps/dashboards/wise-wfd). Waterbodies: https://www.eea.europa.eu/data-and-maps/data/wise-eionet-spatial-2.
**Data quality:** Data gaps occur.
**Attribute information:** Total amount of different hydrographical pressures per 10 x 10 km grid cell.
**Spatial coverage**: Most of mainland Europe and the UK. Data for Norway is missing.
**Spatial resolution**: Polygon features converted to the EEA 10 x 10 km grid.
**Time period and temporal resolution**: 2016

Input of continuous anthropogenic sound

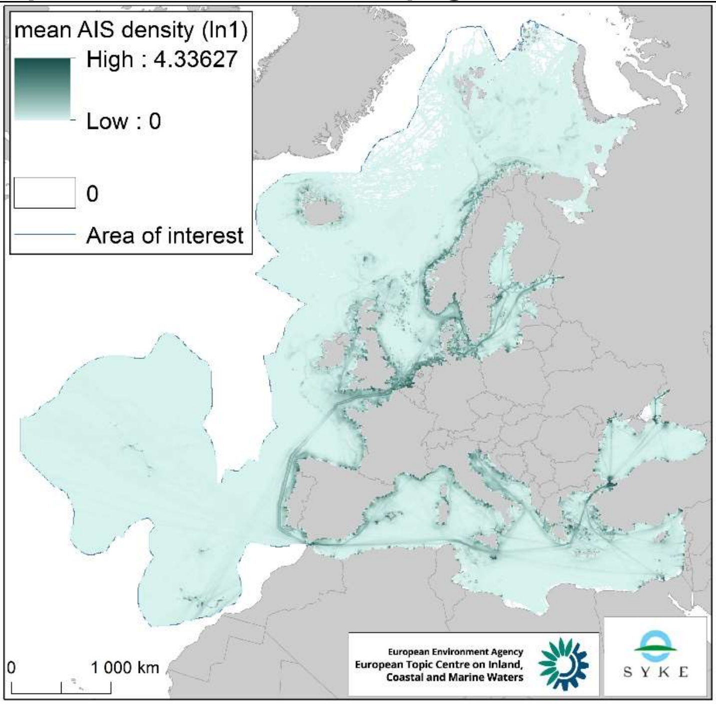

The EMODnet 2017 vessel density dataset was used as a proxy for input of continuous anthropogenic sound. As different vessel types produce different amount of underwater noise, the density of different types was averaged in order to not overestimate noise in areas of high density of small vessels.
The EMODnet data presents the number of hours per month that ships spent in each square 6ilometer in European seas. The vessel density dataset contained very high values at port locations, since ships often idle there with their AIS transponder turned on. To account for this, all values over 100 were truncated to 100. The threshold was selected manually by screening through the data values and comparing to known port locations.

<table>
<tr><td></td><td>Lastly, the dataset was converted to the EEA 10 x 10 km grid by calculating the mean. The dataset was also log transformed (ln+1).
Official EMODnet vessel density metadatadescribes the vessel density layer: https://www.emodnet-humanactivities.eu/documents/Vessel%20density%20maps_method_v1.5.pdf</td></tr>
<tr><td colspan="2">Data source: EMODnet: https://www.emodnet.eu/geoviewer/#!/.</td></tr>
<tr><td colspan="2">Data quality: AIS based vessel density dataset used as proxy for continuous anthropogenic sound.</td></tr>
<tr><td colspan="2">Attribute information: Log transformed monthly average shipping density per 10 x 10 grid cell.</td></tr>
<tr><td colspan="2">Spatial coverage: Area of interest.</td></tr>
<tr><td colspan="2">Spatial resolution: 1 x 1 km grid converted to 10 x 10 km grid, mean.</td></tr>
<tr><td colspan="2">Time period and temporal resolution: Entire 2017.</td></tr>
</table>

<table>
<tr><td colspan="2">Input of impulsive anthropogenic sound</td></tr>
<tr><td>

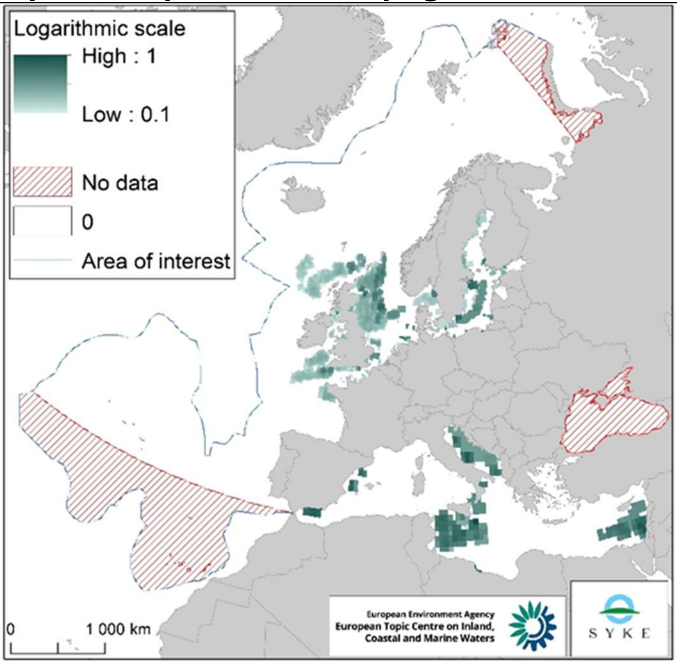

</td><td>The input of impulsive anthropogenic sound layer was created by combining pulse-block-days (PBD) data from the ICES Registry (for HELCOM and OSPAR areas) and ACCOMBAS (for the Mediterranean Sea).
The data available from the ICES register and the ACCOBAMS demonstrator pulse-block-days (PBD) indicator do not give exact locations of impulsive noise emitting activities being carried out (the data shows the number of activities per spatial grid cell of the grid they use).
These data layers were overlaid with the EEA 10 x 10 km grid, which means that information in the grid is not actual pulse-block-days. For example, if cell of the EEA grid has a PBD value of 200, it does not mean that there were 200 PBDs in that 10 x 10 km area, but that 200 PBDs were recorded in a larger area (ICES or ACCOBAMS grid cell) over which this 10 x 10 km area was overlaid. Data is log-tranformed (ln+1).</td></tr>
<tr><td colspan="2">Data source: ICES impusive noise register: https://underwaternoise.ices.dk/impulsive/webservices.aspx, Impulsive noise register for the Mediterranean Sea region: http://80.73.144.60/CTN_Geoportal/home/.</td></tr>
<tr><td colspan="2">Data quality: Data gaps occur. ACCOBAMS data includes data gathered in the study Overview of the Noise Hotspots in the ACCOBAMS Area.</td></tr>
<tr><td colspan="2">Attribute information: Pulse-block-days in area (see text for more details)</td></tr>
<tr><td colspan="2">Spatial coverage: HELCOM and OSPAR areas as well as the Mediterranean Sea. Does not include the Black Sea.</td></tr>
<tr><td colspan="2">Spatial resolution: EEA 10 x 10 km grid.</td></tr>
<tr><td colspan="2">Time period and temporal resolution: 2014 - 2016</td></tr>
</table>

**Input of microbial pathogens**	
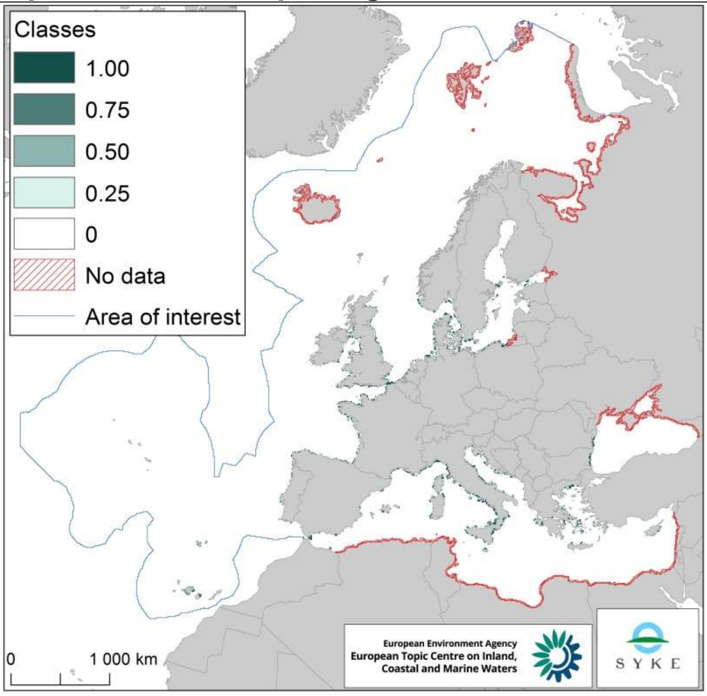	The input of microbial pathogens pressure layer was created using three different datasets converted to the EEA 10 x 10 km grid:<br>1. Urban waste water treatment directive report (urban agglomerations)<br>2. Ports with passenger information<br>3. Intestinal enterococci and Escherichia coli at bathing sites.<br>Urban agglomerations (reported under EU Urban Waste Water Treatment Directive) laying no further than 5 km from the coast were identified and compiled in a common layer. Only agglomerations with more than 5% untreated waste were selected. The information of waste water load (PE) generated by specific agglomerations was used as the proxy for this pressure. Pressure data was extrapolated to the neighbouring cells using 2 buffer belts (6 km-30% reduction) and 12 km-60% reduction. It was finally classified to 0, >0-0.25, >0,25.0.50, >0.50-0.75 and >0.75-1.0.
All ports (EMODnet) lying on the sea coast were selected. Information on number of passengers (annual average 2006-2016) was used as proxy for pressure intensity. Pressure data was extrapolated to the neighbouring cells using buffer belts (6 km-30% reduction) and 12 km-60% reduction. It was finally classified to 0, >0-0.25, >0.25-0.50, >0.50-0.75 and >0.75-1.0.<br>Intestinal enterococci and Escherichia coli data at bathing sites (as measured under the bathing water reporting obligation) gives concentrations of these two bacteria. Pressure value is the average of the two concentrations from the period 2008-2016. It was extrapolated to the neighbouring cells using 5 km buffer belt (50% reduction). The mean was finally classified to 0, >0-0.25, >0,25.0.50, >0.50-0.75 and >0.75-1.0<br>All three datasets were then averaged per grid cell and classified again to the same classes.	
**Data source:** EEA bathing water directive data: https://www.eea.europa.eu/data-and-maps/data/bathing-water-directive-status-of-bathing-water-12, EEA Urban Waste Water Treatment Directive data: https://www.eea.europa.eu/data-and-maps/data/waterbase-uwwtd-urban-waste-water-treatment-directive-7. GISCO Ports 2013: https://ec.europa.eu/eurostat/web/gisco/geodata/reference-data/transport-networks	
**Data quality:** Contains extrapolated data.	
**Attribute information:** Aggregated and classified microbial pathogens pressure.	
**Spatial coverage**:	
**Spatial resolution**: EEA 10 x 10 km grid.	
**Time period and temporal resolution**: UWWTD 2017, Ports 2006-2016 (annual average number of passengers), Bathing sites reporting 2008-2016 (average).	

Input of nutrients

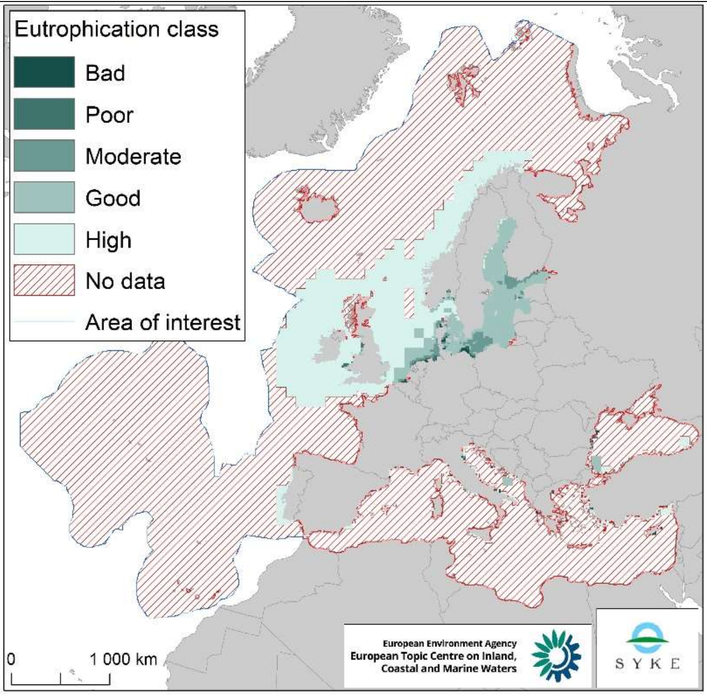

The input of nutrients pressure layer presents the level of eutrophication as an integrated value by the eutrophication assessment tool (HEAT). The layer is prepared by the European Environment Agency (https://www.eea.europa.eu/publications/nutrient-enrichment-and-eutrophication-in). The tool uses all indicators representing nutrient concentrations, direct effects of eutrophication (primary producers, water transparency) and indirect effects of eutrophication (benthic fauna, oxygen). The indicators have thresholds representing disturbed level of eutrophication.

In the HEAT tool, raster cell values over 1 are considered to indicate eutrophication, and values ranging from 0-1 are considered to indicate no significant eutrophication.

While the figure presents all the data, the CEA pressure layer includes only cells with value ≥1 (i.e. eutrophicated areas), and these converted to the EEA 10 x 10 km grid. Additionally, due to some large value outliers, all ≥5 values were truncated to 5.

Data source: Nutrient enrichment and eutrophication in Europe's seas: Moving towards a healthy marine environment (EEA Report No 14/2019, https://www.eea.europa.eu/publications/nutrient-enrichment-and-eutrophication-in).

Data quality: There are wide data gaps in areas beyond continental shelf and all the Mediterranean offshore. According to the EEA report this was due to lack of threshold values to indicators. As the pressure layer represents only areas >1 value (eutrophicated levels), the data gaps do not likely underestimate the pressure in the Mediterranean offshore and NE Atlantic offshore which are oligotrophic environments (Mena et al. 2019). Real pressure gaps are in the NW Black Sea and Adriatic Sea, which are known as eutrophicated areas (Borysova et al. 2005, Strobl et al. 2009). Note that Mediterranean coastal areas are not well visible in the insert map.

Attribute information: Eutrophication ratio per 10 x 10 km grid cell.

Spatial coverage: Northern Europe, coastal Mediterranean, parts of Black Sea.

Spatial resolution: 100 x 100 km grid cells (offshore) and 20 x 20 km grid cells (coast) were converted to the EEA 10 x 10 km grid.

Time period and temporal resolution: HEAT model 2018.

Input of hazardous substances

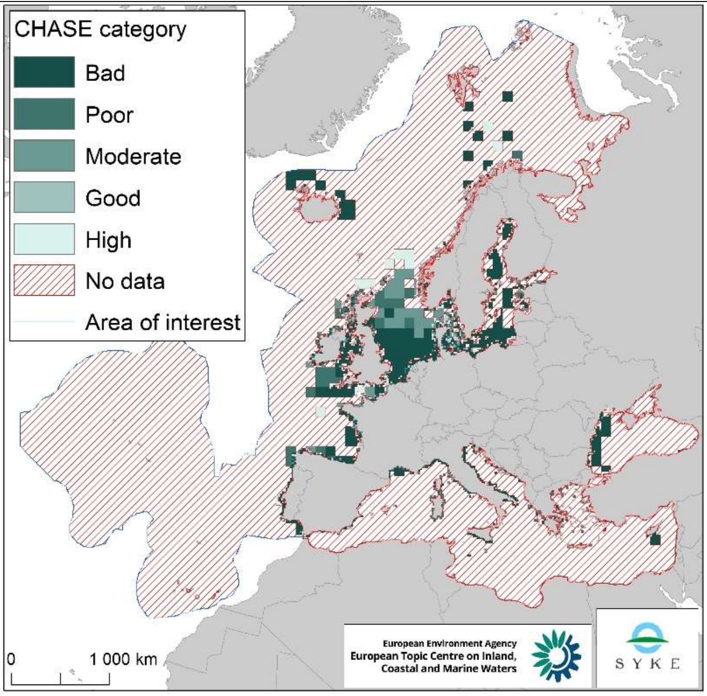

The input of hazardous substances layer presents an integrated layer of substance indicators and polluted areas. Data on EU Priority substances and regionally identified substances were compared with their threshold values using the Hazardous Substances Status Assessment Tool (CHASE; EEA report 25/2018 https://www.eea.europa.eu/publications/contaminants-in-europes-seas). Values ≥1 are considered to indicate contaminated areas, and values ranging from 0- <1 are considered to indicate no significant contamination. While the figure presents all the data, the pressure laye ‘input of hazardous substances’ includes values ≥1 (i.e. contaminated areas), and they converted to the EEA 10 x 10 km grid. Additionally, due to some large value outliers, all >20 values were truncated to 20.

The layer was supported by two additional data sources: locations of ports which are known for their polluted sediments and coastal water bodies where contamination has been identified as a major pressure. These values were assigned to cells not including CHASE data. The GISCO ports point data was converted to the EEA 10 x 10 km grid and value 1 represents the pressure. The WFD polluted water bodies was in table format and listed water bodies where contamination causing activities occur. The table was joined with water body polygon features downloaded from EEA (https://www.eea.europa.eu/data-and-maps/data/wise-eionet-spatial-2). The feature layer was then converted to the EEA 10 x 10 km grid and value 1 represents the pressure.

Lastly, all three datasets were combined into a single 10 x 10 km raster.

Data source: ETC CHASE assessment (https://www.eea.europa.eu/data-and-maps/data/chase-contaminants-assessment), EEA Water Framework Directive reporting (https://www.eea.europa.eu/data-and-maps/dashboards/wise-wfd), GISCO Ports 2013 (https://ec.europa.eu/eurostat/web/gisco/geodata/reference-data/transport-networks).

Data quality: The CHASE layer in the Baltic Sea covers 62 of 215 offshore and 139 of 187 coastal units; in the Black Sea 12 of 111 offshore and 116 of 365 coastal units; in the Mediteranean Sea 80 of 611 offshore and 14 of 1920 coastal units; in the NE Atlantic Ocean 172 of 649 offshore and 888 of 6209 coastal units.

Attribute information: Aggregation (sum) of three datasets.

Spatial coverage: Gaps occur in the North-east Atlantic Ocean.

Spatial resolution: Point, polygon and raster data aggregated to the EEA 10 x 10 km grid.

Time period and temporal resolution: CHASE assessment 2018, WFD Contaminants 2016, ports 2013.

Introductions of non-indigenous species

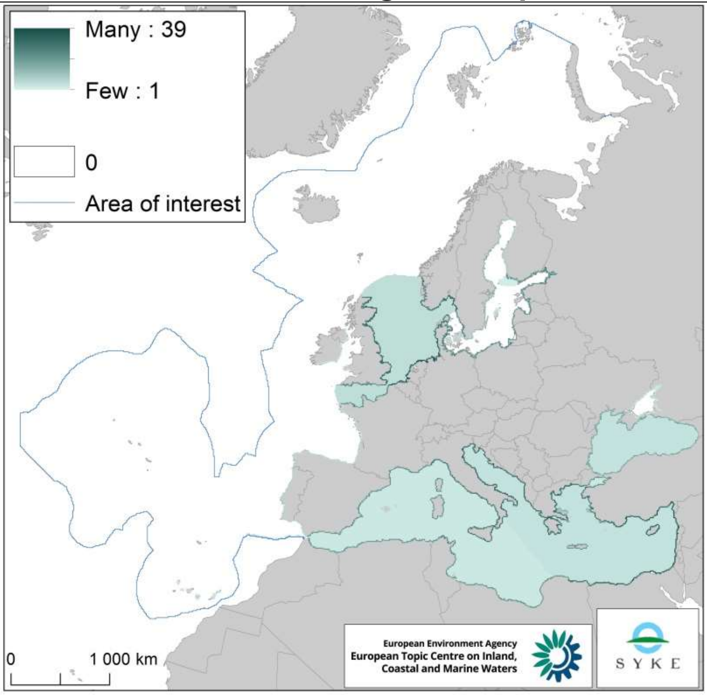

The introductions of non-indigenous species layer presents number of invasive species in EEA 10 km x 10 km grid cells. It was made by first individually mapping distribution areas of each marine invasive species. The distinctive distribution areas for all species were based on previous works such as Katsanevakis et al. 2014) and information from various non-indigenous species online databases:

- Algaebase.org
- Nobanis.org
- Marinespecies.org
- Cabi.org
- Europe-aliens.org
- Ciesm.org
- Invasions.si.edu/nemesis/
- iucn.org
- iucngisd.org/gisd/
- Eol.org
- Marlin.ac.uk

The distribution was mapped according to the information from the aforementioned databases into the EEA 10 x 10 km grid.
For distribution areas of coastal species, only coastal grids (grids outlining the sea areas) were mapped. If the species was observed only occasionally in the area, it was not included. If the species was reported 'often observed' in the area, the area was included in its distribution.
Overall, distribution areas were mapped for 76 different aquatic invasive species. All species layers were summed together into one raster, showing the number of non-indigenous species per 10 x 10 km gird cell.
The dataset was additionally supplemented by non-indigenous species pressure data from Water Framework Directive (WFD) reporting data where non-indigenous species were identified as a pressure the good ecological status in coastal or transitional water bodies. The WFD data was in table format which was joined with water body polygon features, downloaded from EEA (https://www.eea.europa.eu/data-and-maps/data/wise-eionet-spatial-2).
The WFD layer was then compared with the raster composed of the 76 different invasive species (NIS raster). If a cell in the NIS raster had a value of 0 but overlapped with a WFD polygon designated as containing non-indigenous species, the cell was given a value of 1.

Data source: EEA indicator of marine non-indigenous species: https://www.eea.europa.eu/data-and-maps/indicators/trends-in-marine-alien-species-mas-3/assessment.
Water Framework Directive reporting of waterbodies having non-indigeneous species as a pressure: https://www.eea.europa.eu/data-and-maps/dashboards/wise-wfd.
Distribution areas were mapped based on the information from the aforementioned online databases (species observations and regional distribution reports).

Data quality: Some distribution areas were estimated from the reported observations.

Attribute information: Number of non-indigenous species present per 10 x 10 km grid cell.

Spatial coverage: Entire area of interest.

Spatial resolution: Original data was in raster and polygon format, converted to the EEA 10 x 10 grid.

Time period and temporal resolution: 1989 – 2018

Physical disturbance to seabed

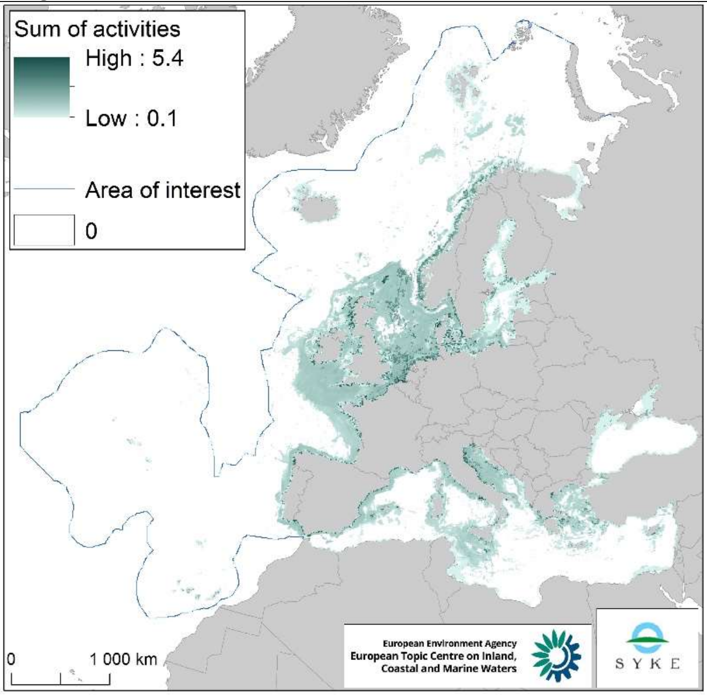

The physical disturbance to seabed layer depicts the sum of all physical disturbance causing activities per EEA 10 x 10 km grid cell.

Data used for *physical disturbance to seabed*:

1. Demersal fishing effort
2. Dredging
3. Sand and gravel extraction
4. Port anchorage sites
5. Windfarms (under construction)
6. Windfarms (partial generation / under construction)
7. Windfarms (decommissioned)
8. Windfarms (operational)

Deposit of dredged matter

9. Oil platforms (offshore installations!)
10. Aquaculture (finfish)
11. Aquaculture (shellfish)
12. Shipping in shallow water

Demersal fishing layer is presented above (‘Bycatch by bottom touching mobile gears’). It was log-transformed and normalized to 0-1. Shipping in shallow waters used the shipping layer is presented above (‘Input of continuous anthropogenic sound’). It was cropped to 0-25 meters depth zone, log-transformed and normalized to 0-1. All the other layers were converted to presence/absence data (value 1 or 0) per 10 x 10 km grid cell.

Physical disturbance was the sum of the values from all the 12 activity layers. Layer for cables and pipes was not used due to poor data quality.

Data source: JRC STECF (see ‘Bycatch by bottom touching mobile gears’), EMODnet https://www.emodnet.eu/geoviewer/#!/, national data portals (e.g. http://marine.gov.scot/, http://www.isde.ie/geonetwork/srv/eng/catalog.search#/home), regional data portals (https://odims.ospar.org/; maps.helcom.fi; https://www.msp-platform.eu/practices/sdimed-geoportal), GISCO (https://ec.europa.eu/eurostat/web/gisco/geodata), 4C Offshore database (purchased.

Data quality: Quality of national data can vary from country to country.

Attribute information: Sum of physical disturbance causing activities per 10 x 10 km grid cell.

Spatial coverage: Entire area of interest.

Spatial resolution: Original data was a mix of feature points and raster files. Finally the data was converted to the EEA 10 x 10 km grid.

Time period and temporal resolution: 2015 for shipping, 2017 for other components

Physical loss of seabed	
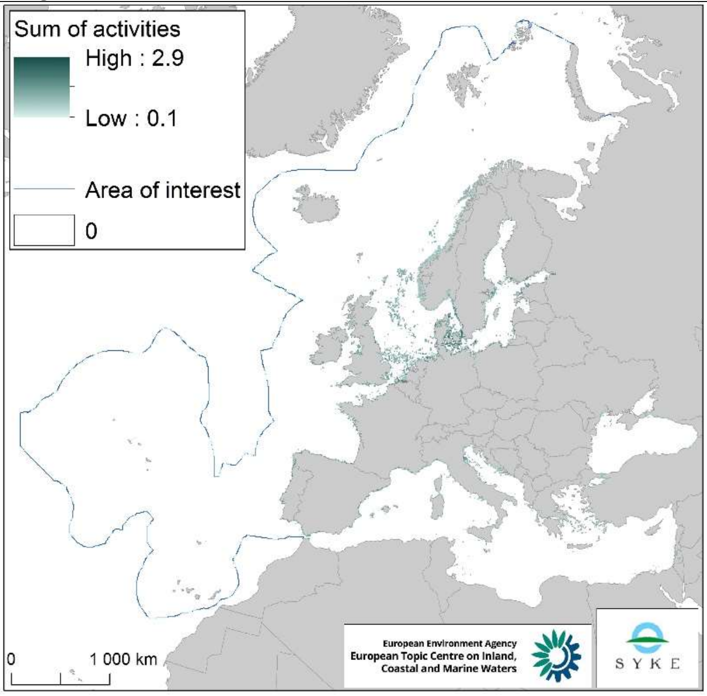	The physical loss of seabed layer depicts the sum of all physical loss causing activities per EEA 10 x 10 km cell.<br>All layers were converted to presence/absence data per 10 x 10 km grid cell before summing them together.<br><br>Data used for physical loss to seabed:<br>1. Dredging<br>2. Dumping of dredged material<br>3. Oil and gas rigs<br>4. Ports<br>5. Sand and gravel extraction<br>6. Operational windfarms
**Data source:** See 'Physical disturbance to seabed'.	
**Data quality:** Quality of national data can vary from country to country.	
**Attribute information:** Sum of physical loss causing activities per 10 x 10 km grid cell.	
**Spatial coverage**: Entire area of interest.	
**Spatial resolution**: Original data was feature point data. Finally the data was converted to the EEA 10 x 10 km grid.	
**Time period and temporal resolution**: 2017	

Sea surface temperature anomalies	
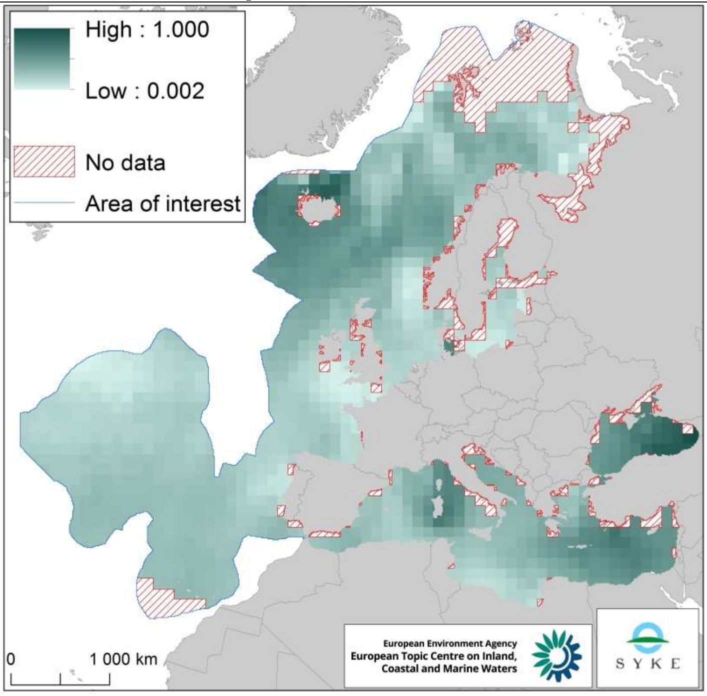	The sea surface temperature (SST) anomalies layer depicts change in SST between 1989 and 2013. The layer was constructed from the data developed to form the EEA product "Mean annual sea surface temperature trend in European seas" (https://www.eea.europa.eu/data-and-maps/figures/mean-annual-sea-surface-temperature-1).<br>The data maps areas of predicted rise of sea surface temperature and also (minor) areas of predicted fall of sea surface temperature. Since all changes of sea temperatures can be considered to have an impact on the marine environment, the pressure layer includes normalised absolute values of SST anomalies.<br>The original data was in a 1° grid format but was converted to the EEA 10 x 10 km grid.
**Data source:** https://www.eea.europa.eu/data-and-maps/figures/mean-annual-sea-surface-temperature-1	
**Data quality:** The SST input data is based on bias adjusted in situ measurements. To extend the analysis over most of the data-sparse oceanic regions, reduced space optimal interpolation was applied (Kaplan et al. 1997).	
**Attribute information:** Absolute value change of sea surface temperature per EEA 10 x 10 km grid cell.	

**Spatial coverage**: Data gaps occur along the coastlines and the northern and southern parts of the area of interest.
**Spatial resolution**: 1° grid cells converted to the EEA 10 x 10 km grid.
**Time period and temporal resolution**: 1989 – 2013

Figure S2: Ecosystem data and description how the spatial habitat and species layers were generated.

Cold-water corals and other coralligenous formations

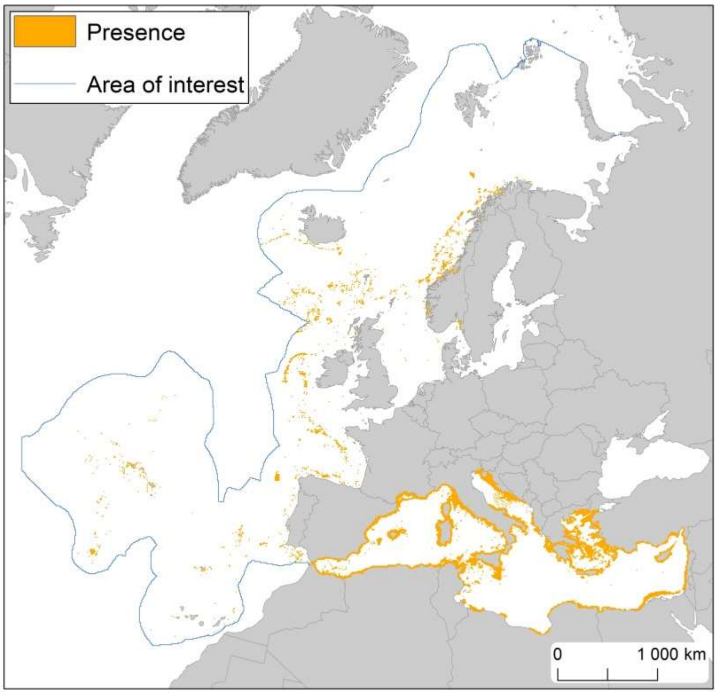

The cold-water corals and other coralligenous formations -layer presents the presence of the habitat in EEA 10 x 10 km grid cells. The layer was created from datasets from two different sources: the UNEP feature dataset on global distribution of cold-water corals and the EMODnet probability of occurrence raster models of coralligenous formations and Maerl in the Mediterranean.

All cells in the EEA 10 x 10 km grid that intersected with the UNEP data were regarded as having a presence of cold-water corals or other coralligenous formations.

Additionally, prediction for over 60 % probability of coralligenous formations or Maerl being present were included to the layer.

Data source: UNEP World Conservation Monitoring Centre: http://data.unep-wcmc.org/datasets/3, EMODnet Seabed habitats for the Mediterranean Sea: https://www.emodnet.eu/en/seabed-habitats.

Data quality: UNEP data is global cold-water coral occurrence data. EMODnet rasters are probability models of coralligenous formations and Maerl in the Mediterranean.

Attribute information: Presence (1) of cold-water corals or other coralligenous formations in 10 x 10 km grid cell.

Spatial coverage: Entire area of interest.

Spatial resolution: Original UNEP data was polygon and point feature. EMODnet models were in 400 x 400 m grids. Both datasets were converted to the EEA 10 x 10 km grid.

Time period and temporal resolution: UNEP 2016-2017, EMODnet 2014.

Saltmarshes distribution

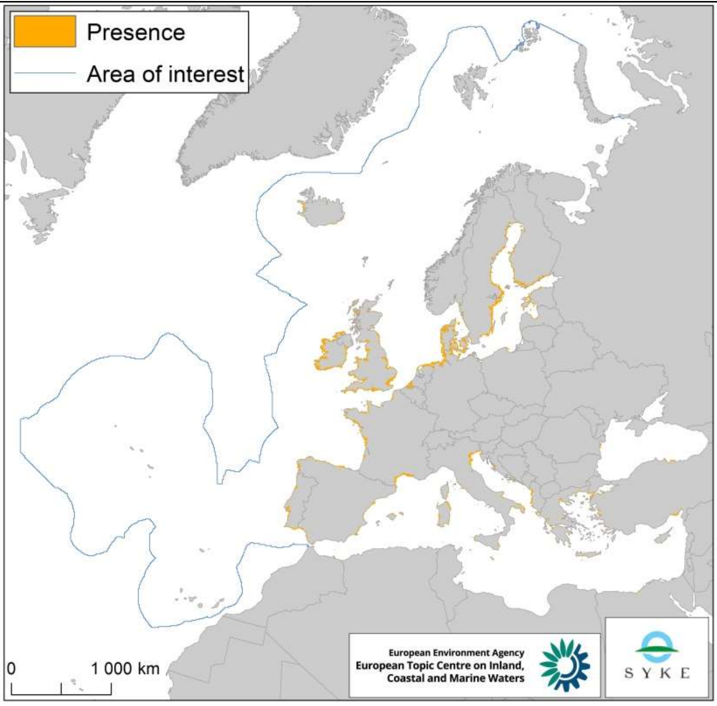

The saltmarshes distribution layer presents the presence of the habitat in EEA 10 x 10 km grid cells.

The layer was created by intersecting the EEA 10 x 10 km grid with data from UNEP feature dataset on global distribution of saltmarshes (http://data.unep-wcmc.org/datasets/43).

All grid cells that intersected at least one saltmarsh feature were designated as having saltmarshes present.

Data source: UNEP World Conservation Monitoring Centre: http://data.unep-wcmc.org/datasets/43

Data quality: Original data was collected using remote sensing and field-based survey methods, with data quality ranging from high-resolution maps to low-resolution representations.

Attribute information: Presence (1) of at least one saltmarsh in 10 x 10 km grid cell.

Spatial coverage: Entire area of interest.

Spatial resolution: Polygon and point data converted to 10 x 10 km grid.

Time period and temporal resolution: 1973-2015

Seagrass distribution

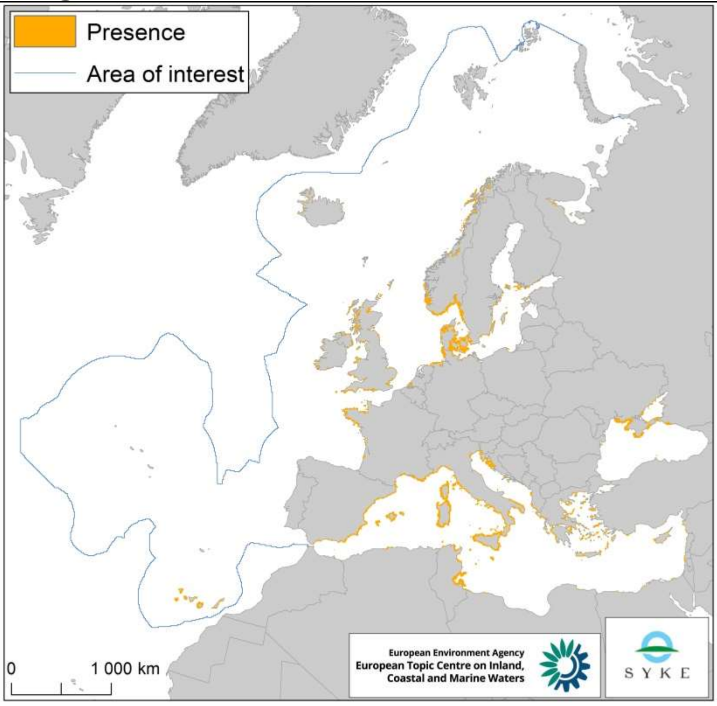

The seagrass distribution layer presents the presence of the habitat in EEA 10 x 10 km grid cells. The layer was created by intersecting the EEA 10 x 10 km grid with the UNEP feature dataset on global distribution of seagrasses (http://data.unep-wcmc.org/datasets/7).

All grid cells that intersected at least one seagrass feature were designated as having seagrass present.

Data source: UNEP World Conservation Monitoring Centre: http://data.unep-wcmc.org/datasets/7

Data quality: Based on occurrence data.

Attribute information: Presence (1) of seagrass in 10 x 10 km grid cell.

Spatial coverage: Entire area of interest.

Spatial resolution: Polygon and point data converted to 10 x 10 km grid.

Time period and temporal resolution: 1934-2015

Breeding sea birds

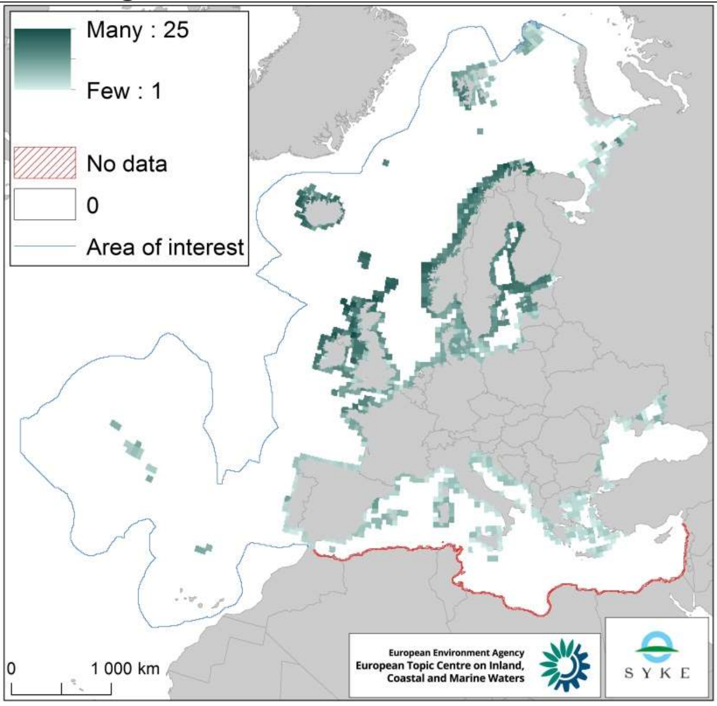

The layer is made from sea bird breeding data provided by the European Breeding Bird Atlas (EBBA).

The original data was in a 50 x 50 km grid used by the European Ornithological Atlas project (EOA), and each grid cell was classified as one of three classes:

0. Non breeding
A. Possible breeding
B. Probable breeding
C. Confirmed

Each bird species had its own entry.

Classes A, B and C were regarded as presence, after which the amount of breeding bird species per EOA grid cell was calculated.

Finally the EOA grid was converted to the EEA 10 x 10 km grid.

Data source: European Breeding Bird Atlas (EBBA v. 1.0): https://www.ebba2.info/

Data quality: Data gaps and uncertainties of species presence occur.

Attribute information: Sum of different breeding sea bird species in each 10 x 10 km cell.

Spatial coverage: Whole are of interest except for the southern and western coasts of the Mediterranean Sea.

Spatial resolution: EOA 50 x 50 km grid converted to 10 x 10 km grid.

Time period and temporal resolution: 01.01.1972 – 31.12.1995

Broad-scale habitats

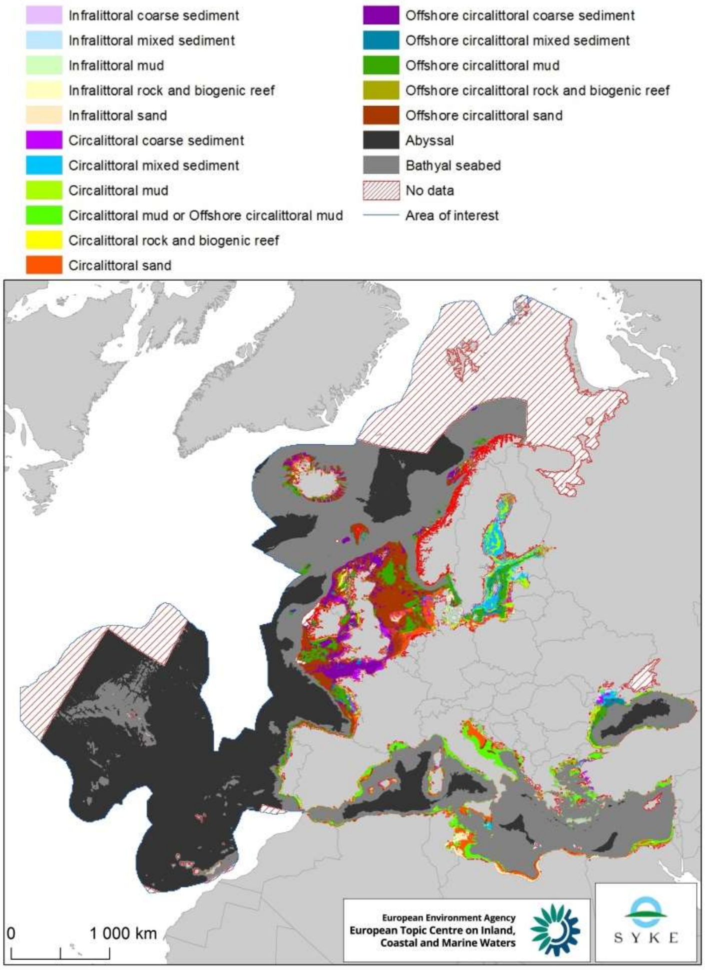

The data is derived from a 2016 broad-scale predictive model produced by EMODnet Seabed Habitats.

For the *broad-scale habitats* ecosystem component, the upper and lower bathyal habitats, of which there originally were a total of 8 different classes, were aggregated into one single class called bathyal seabed.

After the aggregation, 18 different broad-scale habitat classes plus a no data class (NA) remained.

Lastly, a seabed coverage percentage was calculated for each class for each 10 x 10 km grid cell, taking into account the land area of each grid cell.

Broad-scale habitats (after aggregation):

1. Circalittoral coarse sediment
2. Circalittoral mixed sediment
3. Circalittoral mud
4. Circalittoral mud or Offshore circalittoral mud (found only in the Mediterranean)
5. Circalittoral rock and biogenic reef
6. Circalittoral sand
7. Infralittoral coarse sediment
8. Infralittoral mixed sediment
9. Infralittoral mud
10. Infralittoral rock and biogenic reef
11. Infralittoral sand
12. Offshore circalittoral coarse sediment
13. Offshore circalittoral mixed sediment
14. Offshore circalittoral mud
15. Offshore circalittoral rock and biogenic reef
16. Offshore circalittoral sand
17. Bathyal seabed
18. Abyssal seabed

Data source: EMODnet Seabed Habitats: https://www.emodnet.eu/en/seabed-habitats.

Data quality: Gaps occur due to lack of modelled habitat data.

Attribute information: Seabed coverage as a percentage in every 10 x 10 km cell.

Spatial coverage: Data gaps occur in the southwestern and northern parts of the area of interest, as well as the entire Sea of Azov and a small area south of Portugal.

Spatial resolution: The original data was in polygon feature format. Finally the data was converted to the EEA 10 x 10 km grid.

Time period and temporal resolution: The original model was produced in 2016.

**Modelled seamounts distribution**	
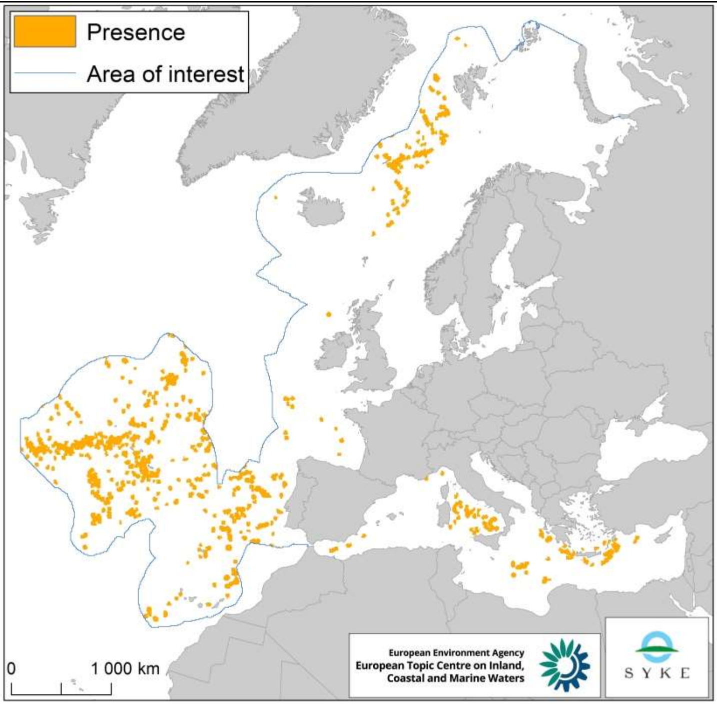	The modelled seamounts distribution layer presents the presence of the habitat in EEA 10 x 10 km grid cells.<br><br>The layer was created by intersecting the EEA 10 x 10 km grid with the UNEP feature dataset on Global Distribution of Seamounts and Knolls (http://data.unep-wcmc.org/datasets/41). Only the data for seamounts were used for the layer.<br><br>All EEA grid cells that intersected with the seamount distribution data were given a value of presence.
**Data source:** UNEP World Conservation Monitoring Centre: http://data.unep-wcmc.org/datasets/41.	
**Data quality:** Seamount locations were inferred, using a searching algorithm, from bathymetric data at 30 arc-sec resolution (SRTM30_PLUS, version 6, which is based on a satellite-gravity model). See Yesson et al. (2011) for full details.	
**Attribute information:** Presence (1) of modelled seamount in 10 x 10 km grid cell.	
**Spatial coverage**: Entire area of interest.	
**Spatial resolution**: Modelled polygon seamounts converted to the EEA 10 x 10 km grid.	
**Time period and temporal resolution**: 2011	

**Fish distribution**	
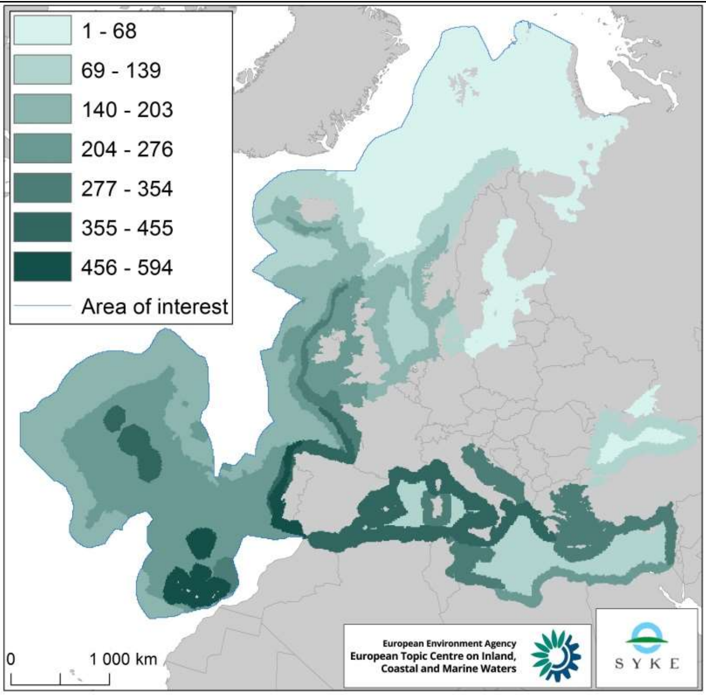	The layer is based on the publication of IUCN European Red List of Marine Fishes (http://cmsdata.iucn.org/downloads/iucn_european_red_list_of_marine_fishes_web_1.pdf).<br><br>The layer presents the number of species per 1000 km2 within the European marine areas. The IUCN data was converted to the EEA 10 x 10 km grid.<br><br>The original dataset did not cover the EEA 10 x 10 km grid in total, but the values were extrapolated to fill in the data gaps.
**Data source:** European Red List of Marine Fishes by IUCN: http://cmsdata.iucn.org/downloads/iucn_european_red_list_of_marine_fishes_web_1.pdf	
**Data quality:** Data is extrapolated on the western part of the Atlantic to cover the whole EEA 10 x 10 km grid. Otherwise the data is European wide.	
**Attribute information:** The number of species per 1000 km2 per 10 x 10 km grid cell.	
**Spatial coverage**: Entire area of interest	
**Spatial resolution**: Original data as areas, converted to EEA 10 x 10 km grid.	

Time period and temporal resolution: Published in 2016

Baleen whales distribution

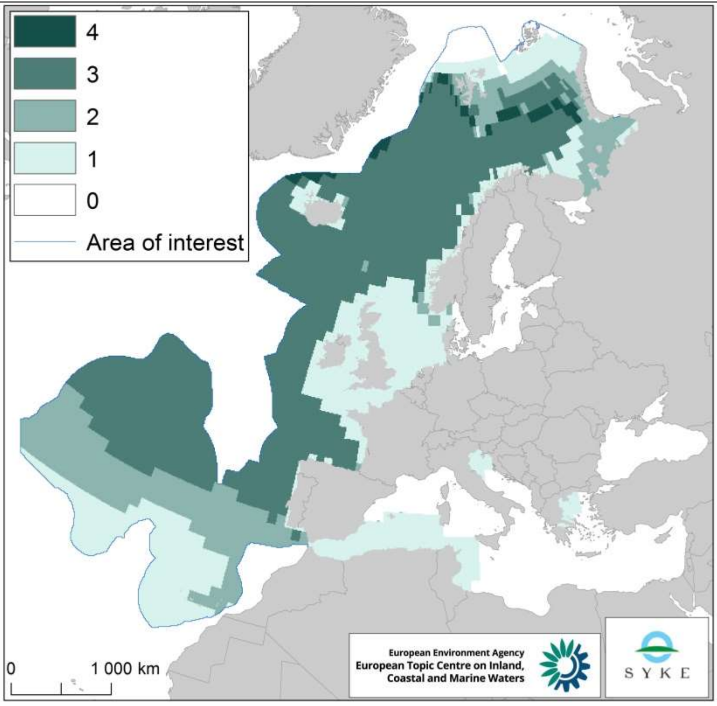

The *Baleen whales distribution* layer estimates the amount of baleen whale species present in each grid cell.

The species represented in the layer are:

1. Bowhead whales
2. Sei whales
3. Humpback whales
4. Blue whales.

The data was derived from probability of occurrence models for above mentioned baleen whales from the Aquamaps portal (https://www.aquamaps.org/). The layer was created by converting each data set to presence/absence data and summing them together within each grid cell. A probability of occurrence of >20% was regarded as presence.

The original data sets were modelled in a 1 ° Ocean Biogeographic Information System (OBIS) Grid. After calculating the sum of bowhead whales, sei whales, humpback whales and blue whales, presence within each OBIS 1 ° grid cell, the grid was converted into the EEA 10 x 10 km grid.

Data source: AquaMaps: https://www.aquamaps.org/.

Data quality: AquaMaps models are expert reviewed.

Attribute information: Sum of baleen whale species present in each 10 x 10 km grid cell.

Spatial coverage: Entire area of interest.

Spatial resolution: Ocean Biogeographic Information System (OBIS) 1° grid converted to the EEA 10 x 10 km grid.

Time period and temporal resolution: Bowhead whale and Sei whale 2013, Humpback whale and Blue whale 2014.

Deep diving toothed cetaceans distribution

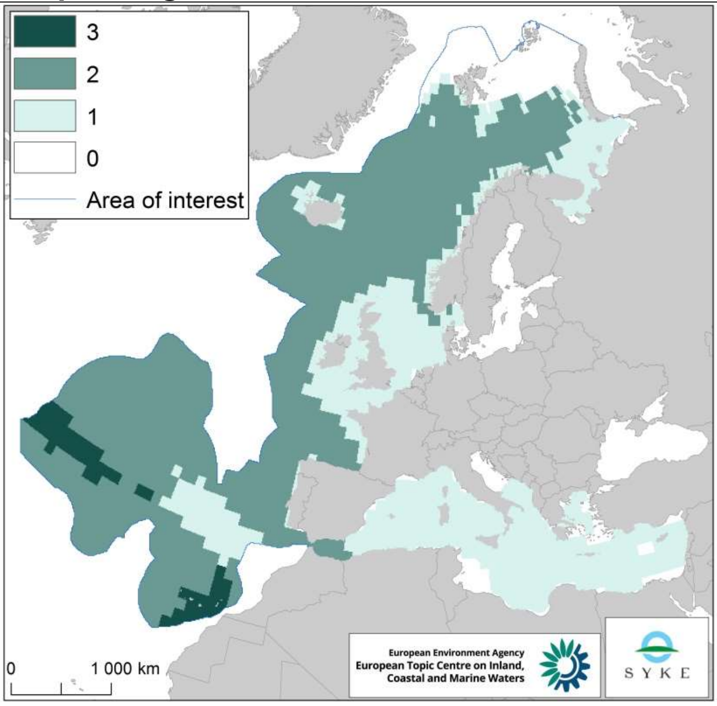

Deep diving toothed cetaceans distribution estimates the amount of deep diving toothed cetaceans species present in each 10 x 10 km grid cell.

The data was derived from probability of occurrence models for sperm whales, northern bottlenose whales and melon-headed whales derived from Aquamaps portal (https://www.aquamaps.org/). The layer was created by converting each data set to presence/absence data and summing them together within each grid cell. A probability of occurrence of >20% was regarded as presence.

The original data sets were in a 1 ° Ocean Biogeographic Information System (OBIS) Grid. After calculating the sum of sperm whale, northern bottlenose whale and melon-headed whale presence within each OBIS 1 ° grid cell, the grid was converted into the EEA 10 x

<table>
<tr><td></td><td>10 km grid.
Species used for deep diving toothed cetaceans distribution:

1. Sperm whale
2. Northern bottlenose whale
3. Melon-headed whale</td></tr>
<tr><td colspan="2">Data source: Aquamaps: https://www.aquamaps.org/.</td></tr>
<tr><td colspan="2">Data quality: Gaps occurs due to lack of modelled mammal data. Models are expert reviewed.</td></tr>
<tr><td colspan="2">Attribute information: Sum of the number species present in cell.</td></tr>
<tr><td colspan="2">Spatial coverage: Entire area of interest.</td></tr>
<tr><td colspan="2">Spatial resolution: Ocean Biogeographic Information System (OBIS) 1° grid converted to the EEA 10 x 10 km grid.</td></tr>
<tr><td colspan="2">Time period and temporal resolution: Mammal models were produced in 2013.</td></tr>
</table>

<table>
<tr><td colspan="2">Seal distribution</td></tr>
<tr><td>

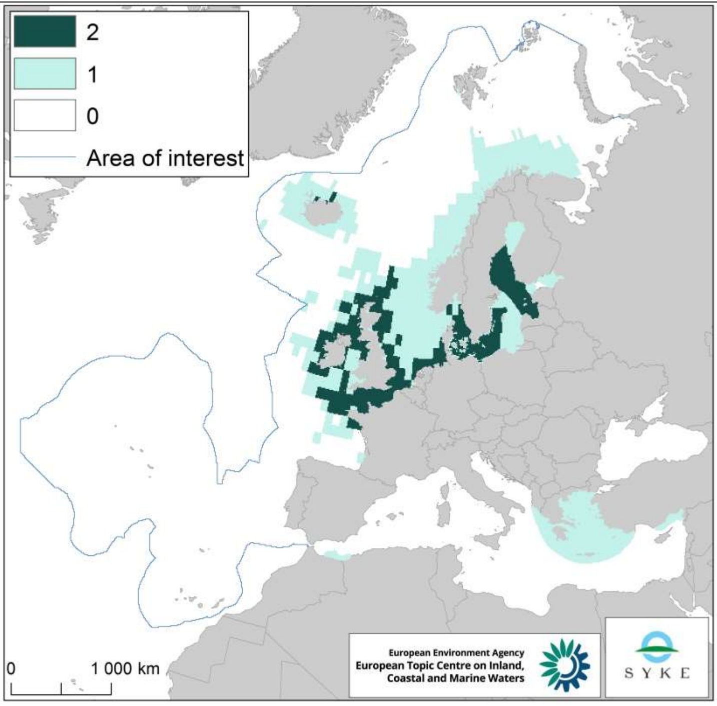

</td><td>Seal distribution estimates the amount of seal species present in each 10 x 10 km grid cell.

The layer was created from a mix of modelled probability of occurrence, sightings, and distribution area data (see Data source).

Modelled or sighting data (Harbour seal and Grey seal) was first converted to presence/absence per Ocean Biogeographic Information System (OBIS) grid cell (1°) and then resampled to 10 x 10 km grid cells. For modelled probability of occurrence data (Grey seal), a probability of >20% was regarded as presence.
The distribution areas for Ringed seals, Harbour seals and Monk seals were straight converted to presence in the EEA 10 x 10 km grid.

Lastly the data was summed together for the final result.
Species used for seal distribution:
1. Grey seal
2. Harbour seal
3. Ringed seal
4. Monk seal</td></tr>
<tr><td colspan="2">Data source: AquaMaps (https://www.aquamaps.org/), Ocean Biogeographic Information System (OBIS; https://obis.org/), HELCOM (maps.helcom.fi), IUCN Marine mammals and sea turtles of the Mediterranean and Black Seas (https://www.iucn.org/content/marine-mammals-and-sea-turtles-mediterranean-and-black-seas).</td></tr>
<tr><td colspan="2">Data quality: Harbour seal OBIS sightings data was supplemented with HELCOM occurrence data for the Baltic Sea. Monk seal OBIS sightings data was supplemented with IUCN occurrence data for the Mediterranean. AquaMaps probability model for grey seal is expert reviewed.</td></tr>
<tr><td colspan="2">Attribute information: Sum of seals present in each 10 x 10 km grid cell.</td></tr>
<tr><td colspan="2">Spatial coverage: Entire area of interest.</td></tr>
<tr><td colspan="2">Spatial resolution: Ocean Biogeographic Information System (OBIS) 1° grid converted to the EEA 10 x 10 km grid.</td></tr>
<tr><td colspan="2">Time period and temporal resolution: AquaMaps grey seal probability of occurrence model 2013. HELCOM harbour seal and ringed seal distribution area models 2013. IUCN monk seal distribution area model 2012. Harbour seal and monk seal sightings data from years 1990-2016.</td></tr>
</table>

Small toothed cetaceans distribution

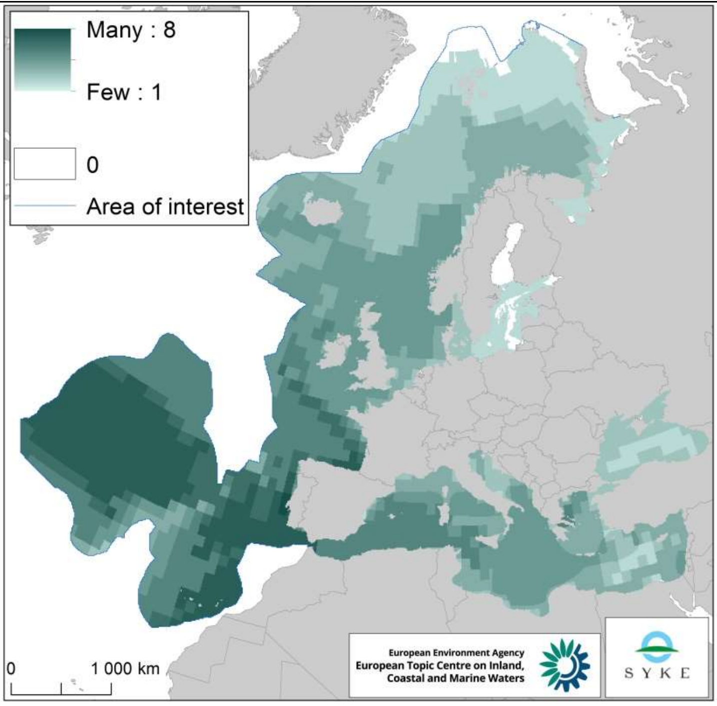

Small toothed cetaceans distribution estimates the amount of small toothed cetacean species present in each 10 x 10 km grid cell.
The layer was created from modelled probability of occurrence from [AquaMaps (https://www.aquamaps.org/)](https://www.aquamaps.org/).
This data was supplemented with modelled harbour porpoise probability of occurrence data from the SAMBAH project in the Baltic Sea, and common dolphin distribution area data from IUCN in the Mediterranean Sea.
For AquaMaps modelled probability of occurrence data, a probability of >20% was regarded as presence. All modelled probability data was first converted to presence/absence per Ocean Biogeographic Information System (OBIS) grid cell (1°) and then resampled to EEA 10 x 10 km grid cells.
For the SAMBAH modelled probability of occurrence data, a probability of occurrence of >0% was regarded as presence.
The IUCN Mediterranean Sea data was directly converted to the EEA 10 x 10 km grid as presence/absence.
Lastly the layers were summed together for the final result, which indicates the amount of small toothed cetacean species present in each grid cell.
Species used for *small toothed cetaceans distribution*:

1. Atlantic spotted dolphin
2. Harbour porpoise
3. Common doplhin
4. Bottlenose dolphin
5. Striped dolphin
6. Orca
7. Long-finned pilot whale
8. Short-finned pilot whale

Data source: AquaMaps (https://www.aquamaps.org/), SAMBAH project (maps.helcom.fi), IUCN Marine mammals and sea turtles of the Mediterranean and Black Seas (https://www.iucn.org/content/marine-mammals-and-sea-turtles-mediterranean-and-black-seas), Ocean Biogeographic Information System (OBIS, obis.org).

Data quality: AquaMaps models are expert reviewed.

Attribute information: Sum of small toothed cetacean species present in each 10 x 10 km grid cell.

Spatial coverage: Entire area of interest.

Spatial resolution: Ocean Biogeographic Information System (OBIS) 1° grid converted to the EEA 10 x 10 km grid.

Time period and temporal resolution: AquaMaps 2009-2018. SAMBAH harbour porpoise model was produced in 2017. IUCN common dolphin data was produced in 2012.

Coastal water column habitat

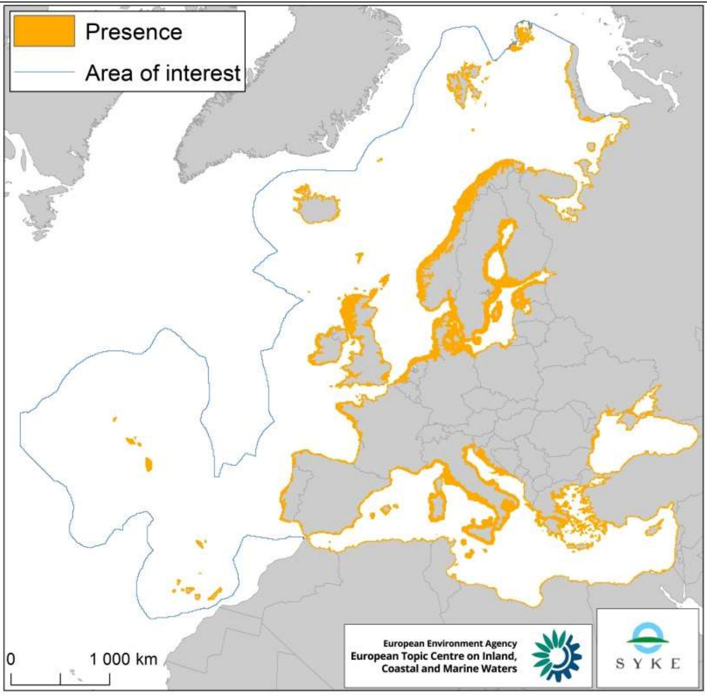

The coastal water column habitat layer presents the presence of the habitat in EEA 10 x 10 km grid cells.

The data was created using the Water Framework Directive Water Bodies polygon dataset.

All EEA 10 x 10 km grid cells that intersected with a waterbody were designated as coastal water column habitats.

Some coastal areas were not featured in the WFDW data. In these cases, all EEA grid cells that intersected with the coast line were regarded as coastal water column habitats. Thematic Mapping's World Borders Dataset was used as coastline data.

Data source: Water Framework Directive Water Bodies: https://www.eea.europa.eu/data-and-maps/data/wise-eionet-spatial-2.

Data quality: Quality assured by European member states.

Attribute information: Presence (1) of coastal water column habitat.

Spatial coverage: Entire area of interest

Spatial resolution: The source data was in polygon feature format. Finally the data was converted to the 10 x 10 km grid.

Time period and temporal resolution: 2014 - 2016

Offshore water column habitat

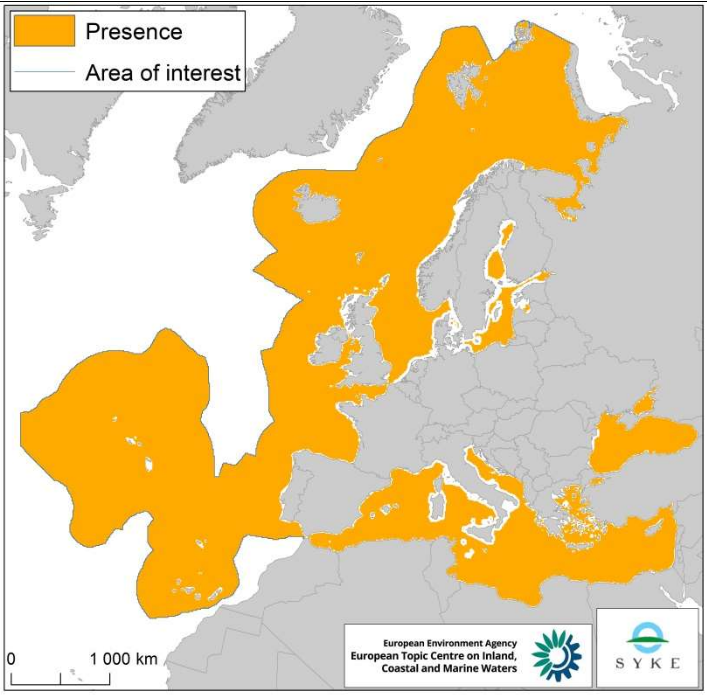

The offshore water column habitat layer presents the presence of the habitat in EEA 10 x 10 km grid cells.

The data was created using the Marine area from European Environment Agency and Water Framework Directive Water Bodies (WFD) polygon dataset.

All EEA 10 x 10 km grid cells that did not intersect with a waterbody were designated as offshore water column habitats.

Some coastal areas were not featured in the WFDW data. In these cases, all EEA grid cells that did not intersect with the coast line were regarded as offshore water column habitats. Thematic Mapping's World Borders Dataset was used as coastline data.

Data source: Marine area: https://www.eea.europa.eu/data-and-maps/data/europe-seas. Water Framework Directive Water Bodies: https://www.eea.europa.eu/data-and-maps/data/wise-eionet-spatial-2. Thematic Mapping's World Borders Dataset was used as coastline data (https://thematicmapping.org/downloads/world_borders.php).

Data quality: WFDW data supplemented with coastline data.

Attribute information: Presence (1) of offshore water column habitat.

Spatial coverage: Entire area of interest

**Spatial resolution**: The source data was in polygon feature format. Finally the data was converted to the 10 x 10 km grid.
**Time period and temporal resolution**: 2014 - 2016

Turtles distribution

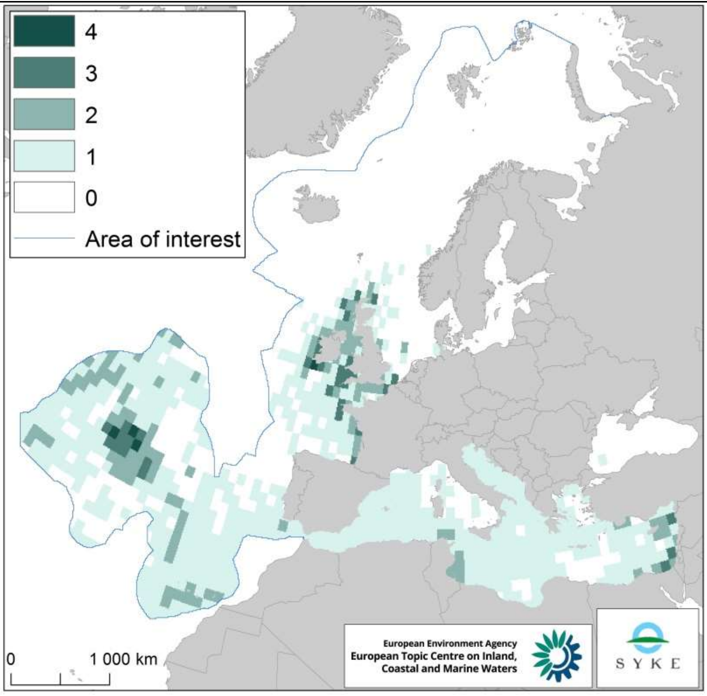

The turtles distribution layer estimates the amount of turtle species present in each 10 x 10 km grid cell.

The layer was created by calculating how many different turtle species were present within each Ocean Biogeographic Information System (OBIS) 1° grid cell. Finally the grid was resampled to the EEA 10 x 10 km grid.

Species used for *turtles distribution*:

1. Loggerhead turtle
2. Green turtle
3. Hawksbill turtle
4. Kemp's ridley turtle
5. Leatherback turtle

**Data source:** Ocean Biogeographic Information System (OBIS): http://www.iobis.org/.
**Data quality:** Original data contains a mix of sightings and presence data in OBIS 1° grid.
**Attribute information:** Sum of turtle species present in each 10 x 10 km grid cell.
**Spatial coverage**: Entire area of interest.
**Spatial resolution**: Ocean Biogeographic Information System (OBIS) 1° grid converted to 10 x 10 km grid.
**Time period and temporal resolution**: Sightings data and presence data from years 1990-2016.

Appendix S2. Survey results for ecosystem sensitivity to anthropogenic pressures.

Estimation of the ecosystem sensitivity to each of the pressures was surveyed among European marine experts. The expert survey was distributed through the European Topic Centre for Inland, Coastal and Marine waters (ETC-ICM) for experts in three European marine regions: Black Sea, Mediterranean Sea and NE Atlantic Ocean. In the Baltic Sea, a similar survey was carried out very recently and the results were available for this study (HELCOM 2018).

The survey was made as an Excel template with the following content: guidance page (see Figure S3), contact information, selection of marine region for responses, list of pressures and ecosystem, components in the survey, and the survey matrix with pre-filled drop-down menus. The survey asked for each combination of pressures and ecosystem components the ‘sensitivity’ (0. No effect, 1. Very low, 2. Low, 3. Moderate, 4. High, 5. Very high). In addition, the survey

Each marine region had a contact point (part of the author team) who sent out the questionnaire to senior experts of marine ecosystem. The contact points were available for questions and advice for filling the survey. It was emphasized that not all questions need to be answered. In total, 45 experts responded to the survey from the Mediterranean Sea (29), NE Atlantic Ocean (11) and Black Sea (5). In addition to that, results from 81 experts in nine countries in the Baltic Sea were included to the survey.

The sensitivity estimates were analyzed between the regions to see if there is statistical evidence to consider the estimates similar and combine them for the analysis. The test was done by both the Savage and the Sigel-Tukey non-parametric tests because the sensitivity scores are in ordinal scale. As 87 % of the sensitivity estimates did not differ according to the tests (the stricter of the two tests was selected), we combined the values by taking median values over the four regions. Tables S2-S5 present the regional median values for the sensitivities and Table S6 presents test results for their differences.

In order to understand variability of the expert responses, we also calculated means and standard deviations (SD) of the values, by assuming that the sensitivity scores from 0 to 5 are in interval scale. Less than 1% of sensitivity scores had SD>2, 30% had SD 1.5-2, 62% had SD 1.0-1.5 and 7% had SD <1.0. Highest variability (SD>2) was found for sensitivity of abyssal habitats to *physical loss*, saltmarshes to *physical loss* and seagrasses to *bycatch by bottom-touching mobile gears*.

Figure S3. Guidance page to the expert survey for sensitivity of ecosystem components to pressures.

Guidance for the expert survey on setting of impact scores

The survey is filled to one MSFD sea region at time. You can inform on the next sheet which region to respond to. Another questionnaire can be filled for another sea region.

The questionnaire is on the 3rd sheet. In this, you are encouraged to fill the entire questionnaire as an expert of marine ecology. In cases where you are highly uncertain about the answer, you can leave a blank space.

Sensitivity. When answering the questionare, you will have to fill in information on how sensitive the listed ecosystem components (e.g. seagrasses or baleen whales) are for different man made pressures (e.g. nutrient inputs, extraction of species, physical disturbance of seabed). In this, you should primarily consider direct effects from the pressures. In case of species, you should consider the population effects, not an individual effect.

This information will contribute to a data set, that will facilitate an estimation of the ecological vulnerability of ecosystem components to certain pressures. The last sheet includes some comments about the ecosystem components.

On the next spread sheet, you will be asked to give some basic personal information. This information will be used exclusively to contact you about the results. We guarantee that your personal information will not be used for any other purposes or given to anyone outside the core project team.

The survey consist of a matrix like this:

Sensitivity

	**Circalittoral coarse sediment**	**Seals**
Physical disturbance to seabed		
Input of nutrients		
Input of underwater noise		

The Ecosystem components are listed in the top row and the pressures are listed in the first column.
For each combination of ecosystem components and pressures, please fill in the sensitivity of the of the ecosystem component to the pressure.

Detailed information and definitions:
Sensitivity is defined as the tolerance of an ecosystem component against a pressure (i.e. tolerate without being adversely affected) and the recoverability of it after an adverse effect. In non-continuous pressures (e.g. dredging, fishing) the recoverability is an important aspect of sensitivity. In continuous pressures (e.g. nutrient concentration, inputs of microbial pathogens), the system does not recover and the sensitivity is considered by the tolerance factor. In the questionnaire, you should consider sensitivity against a relatively high pressure intensity.

Filling the questionnaire:

Sensitivity Choose from the dropdown menu or write:

"N" or "0" for "No effect"
"VL" or "1" for "Very low"
"L" or "2" for "Low"
"M" or "3" for "Moderate"
"H" or "4" for "High"
"VH" or "5" for "Very high"

Table S2. Ecosystem sensitivity estimates in the Baltic Sea. The pressures and ecosystem components are ranked by their sensitivity (median of the survey responses) from left and up (higher sensitivity) to right and down (lower sensitivity).

	Fish species	Coastal water column habitat	Infralittoral rock and biogenic reef	Circalittoral rock and biogenic reef	Seagrass	Infralittoral mud	Circalittoral sand	Infralittoral sand	Infralittoral mixed sediment	Breeding sea birds	Small toothed cetaceans	Infralittoral coarse sediment	Circalittoral mud	Circalittoral coarse sediment	Saltmarshes	Offshore circalittoral rock and biogenic reef	Seals	Offshore circalittoral mud	Circalittoral mixed sediment	Offshore water column habitat	Offshore circalittoral sand	Offshore circalittoral coarse sediment	Offshore circalittoral mixed sediment
Physical loss of seabed	3	2	4	5	5	4	5	4	5	1	1	4	3,5	5	5	5	1	3,3	5	1	3,3	2,8	3,3
Sea surface temperature anomalies	3	4	4	3,5	4	3	3	3	3	3	3	3	3	3	4	3	3	3	3	3	3	3	2,5
Inputs of hazardous substances	4	3	3	3	3	3	3	3	3	4	4	3	3	3	3	3	4	3	3	2	3	3	3
Physical disturbance to seabed	3	2,8	4	4	4	4	3	4	3	1	1	3	3	3	4	3,3	1	4	3	0,6	3	3	3
Bycatch by bottom touching mobile gears	4	2	3	4	2	3	4	3	3	1	1,5	3	4	4	0	3	1	4	4	1	4	4	4
Input of nutrients	3	4	4	3	4	3	3	3	3	2	1	3	2,5	2,5	4	3	1	2	2	4	2	2	2
Extraction of species by commercial fishing	4,6	3	3	4	2	2	3	2	2	2,5	2	2	4	3	0	3	2	4	1	3	4	1	1
Introductions of non-indigenous species	3	3	3	3	3	3	2,8	3	3	1	1	3	2,	3	3	2	0	2	3	2	2	2	2
Changes to hydrological conditions	3	3	3	2	4	3	2	3	2,4	1	0,05	3	2	2	3	2	1	2	2	1,5	1	2	2
Input of microbial pathogens	3	2,5	2	2	2	2	2	2	2	3,5	2	2	2	2	2	1	3,5	1	2	2	1	1	1
Disturbance of species due to human presence	2,1	2	2	2	3,5	2	2	2	2	4	3	2	1	1	2,8	1	3,1	1	1	0	0,5	1	1
Input of continuous anthropogenic sound	3	2	1	1	0	1	1	0,8	0,8	3	4	0,8	1	0	0	1	3	0	0	2	0	0	0
Bycatch by pelagic towed gears	4	3	1	0	0	1	0	1	1	3	3,9	0,5	0	0	0	0	3	0	0	3	0	0	0
Input of impulsive anthropogenic sound	3	2	1	1	0	0	0	0	0	3	5	0	0	0	0,5	0,9	4	0	0	2	0	0	0

Table S3. Ecosystem sensitivity estimates in the Black Sea. The pressures and ecosystem components are ranked by their sensitivity (median of the survey responses) from left and up (higher sensitivity) to right and down (lower sensitivity).

	Fish species	Seagrass	Infralittoral rock and biogenic reef	Saltmarshes	Coastal water column habitat	Infralittoral mud	Infralittoral sand	Infralittoral mixed sediment	Infralittoral coarse sediment	Small toothed cetaceans	Turtles	Circalittoral rock and biogenic reef	Circalittoral sand	Circalittoral mud	Breeding sea birds	Offshore circalittoral mud	Circalittoral coarse sediment	Offshore circalittoral coarse sediment	Circalittoral mixed sediment	Offshore circalittoral mixed sediment	Offshore circalittoral rock and biogenic reef	Offshore circalittoral sand	Offshore water column habitat	Bathyal seabed	Abyssal seabed
Extraction of species by commercial fishing	5	2	3	3	3	5	5	5	5	4,5	4	4	3	4	2,5	4	3	5	3	5	3	4	3	3	2
Sea surface temperature anomalies	3	4	4	4	4	3	3	3	3	3	4	3,5	3	3	3	3	3	3	3	2,5	3	3	3	2	1,5
Bycatch by bottom touching mobile gears	4	5	3	0	2	3	3	3	3	1,5	2	4	4	4	1	4	4	4	4	4	3	4	1	5	2
Inputs of hazardous substances	4	3	3	3	3	3	3	3	3	4	4	3	3	3	4	3	3	3	3	3	3	3	2	2	2
Physical loss of seabed	3	5	4	5	2	4	4	3,3	4	1	2	2,4	3,3	3,5	0,8	3,3	2,4	2,8	2,8	3,3	1,9	3,3	1	2	1
Physical disturbance to seabed	2	4	4	4	2,8	4	4	3	3	1	1	4	3	3	0,5	4	3	3	3	3	3,3	3	0,6	1,5	1
Input of nutrients	3	4	4	4	4	3	3	3	3	1	1,5	3	3	2,5	2	2	2,5	2	2	2	3	2	1	1	0,6
Changes to hydrological conditions	3	4	4	5	3	3	3	4	3	0,05	1	2	2	2	1	2	2	2	2	2	2	1	1,5	1	1
Introductions of non-indigenous species	3	3	3	3	3	3	3	3	3	1	1	2	2,8	2,6	1	2	3	2	3	2	2	2	2	0	0
Disturbance of species due to human presence	2	3,5	4	5	2	2	2	2	2	3	3	2	2	1	4	1	1	1	1	1	1	0,5	0	0	0
Input of microbial pathogens	3	2	2	4	2,5	2	2	2	2	2	0,6	2	2	2	0,8	1	2	1	2	1	1	1	2	0	0
Bycatch by pelagic towed gears	4	2	1	0	3	1	1	1	0,5	3,9	4	0	0	0	3	0	0	0	0	0	0	0	3	1	0
Input of continuous anthropogenic sound	3	0	1	0	2	1	0,8	0,8	0,8	4	3	1	1	1	3	0	0	0	0	0	1	0	2	0	0
Input of impulsive anthropogenic sound	3	0	1	0,5	2	0	0	0	0	5	3	1	0	0	3	0	0	0	0	0	0,9	0	2	0	0

Table S4. Ecosystem sensitivity estimates in the Mediterranean Sea. The pressures and ecosystem components are ranked by their sensitivity (median of the survey responses) from left and up (higher sensitivity) to right and down (lower sensitivity).

	Fish species	Infralittoral rock and biogenic reef	Seagrass	Coastal water column habitat	Turtles	Circalittoral rock and biogenic reef	Saltmarshes	Cold-water corals and other coralligenous formations	Infralittoral mixed sediment	Infralittoral sand	Breeding sea birds	Infralittoral mud	Seals	Small toothed cetaceans	Infralittoral coarse sediment	Circalittoral sand	Circalittoral mud	Deep diving toothed cetaceans	Circalittoral mixed sediment	Circalittoral coarse sediment	Offshore circalittoral mud	Offshore circalittoral rock and biogenic reef	Offshore circalittoral coarse sediment	Offshore circalittoral mixed sediment	Offshore circalittoral sand	Baleen whales	Offshore water column habitat	Seamounts	Bathyal seabed	Abyssal seabed
Extraction of species by commercial fishing	5	3	2	3	4	4	2	3	4	4	2,5	3	4	4	3,5	3	4	2	4	3	4	3	4	4	4	2	3	3	3	2
Sea surface temperature anomalies	3	4	4	4	4	3,5	4	4	3	3	3	3	3	3	3	3	3	3	3	3	3	3	3	2,5	3	3	3	3	2	1,5
Inputs of hazardous substances	4	3	3	3	4	3	3	2,5	3	3	4	3	4	4	3	3	3	4	3	3	3	3	3	3	3	3,5	2	2	2	2
Bycatch by bottom touching mobile gears	4	3	5	2	2	4	0	4	3	3	1	3	1	1,5	3	4	4	1,5	4	4	4	3	4	4	4	1,5	1	3	4	2
Physical loss of seabed	3	4	5	2	2	4	5	4,1	4	4	3	4	1	1	4	3	3,5	0,6	3	3	3,3	3	2,8	3,3	3,3	0	1	4	2	1
Physical disturbance to seabed	4	4	4	2,8	1	4	4	4	3	4	3	4	1	1	3	3	3	1	3	3	4	3,3	3	3	3	0	0,6	3	1,5	1
Input of nutrients	3	4	4	4	1,5	3	4	3	3	3	2	3	1	1	3	3	2,5	1	2	2,5	2	3	2	2	2	1	2	1	1	0,6
Changes to hydrological conditions	3	4,5	4	3	1	2	5	3	4	3	1	3	1	0,1	3	2	2	1	2	2	2	2	2	2	1	1	1,5	1	1	1
Introductions of non-indigenous species	3	4	3	3	1	4	3	3,5	3	3	1	3	0	1	3	2,8	2,6	0	3	3	2	2	2	2	2	0	2	1	0	0
Disturbance of species due to human presence	2,1	4	3,5	2	3	2	4	2	2	2	4	2	5	3	2	2	1	3	1	1	1	1	1	1	0,5	2	0	0	1	0
Input of microbial pathogens	3	2	2	2,5	4	2	3	2	2	2	2	2	3,5	2	2	2	2	2	2	2	1	1	1	1	1	1,5	2	0	0	0
Bycatch by pelagic towed gears	4	1	2	3	4	0	0	1	1	1	3	1	3	3,9	0,5	0	0	3	0	0	0	0	0	0	0	2	3	1	1	0
Input of impulsive anthropogenic sound	3	1	0	2	3	1	0,5	1	0	0	3	0	4	5	0	0	0	5	0	0	0	0,9	0	0	0	5	2	0	0	0
Input of continuous anthropogenic sound	3	1	0	2	3	1	0	0	0,8	0,8	3	1	3	4	0,8	1	1	4	0	0	0	1	0	0	0	4	2	0	0	0

Table S5. Ecosystem sensitivity estimates in the NE Atlantic Ocean. The pressures and ecosystem components are ranked by their sensitivity (median of the survey responses) from left and up (higher sensitivity) to right and down (lower sensitivity).

	Fish species	Coastal water column habitat	Cold-water corals and other coralligenous formations	Infralittoral rock and biogenic reef	Circalittoral rock and biogenic reef	Seagrass	Infralittoral mud	Turtles	Infralittoral sand	Small toothed cetaceans	Infralittoral coarse sediment	Circalittoral mud	Infralittoral mixed sediment	Deep diving toothed cetaceans	Circalittoral sand	Offshore circalittoral rock and biogenic reef	Saltmarshes	Breeding sea birds	Circalittoral coarse sediment	Offshore circalittoral mud	Seals	Circalittoral mixed sediment	Offshore circalittoral coarse sediment	Offshore circalittoral sand	Offshore circalittoral mixed sediment	Offshore water column habitat	Baleen whales	Seamounts	Bathyal seabed	Abyssal seabed
Sea surface temperature anomalies	3	4	4	4	3,5	4	3	4	3	3	3	3	3	3	3	3	4	3	3	3	3	3	3	3	2,5	3	3	3	2	1,5
Inputs of hazardous substances	4	3	2,5	3	3	3	3	4	3	4	3	3	3	4	3	3	3	4	3	3	4	3	3	3	3	2	3,5	2	2	2
Extraction of species by commercial fishing	4,5	3	3	3	4	2	3,5	4	3	3	3	4	3	2	3	3	0,5	2,5	3	4	3	2,5	3	4	2,5	3	2	3	3	2
Physical loss of seabed	3	2	4,1	4	4	5	4	2	4	1	4	3,5	2,5	0,6	2	4	5	0,8	2,9	3,3	1	2,3	2,8	3,3	3,3	1	0	4	2	1
Bycatch by bottom touching mobile gears	4	2	4	3	4	1,5	3	2	3	1,5	3	4	3	1,5	4	3	0	1	4	4	1	4	4	4	4	1	0,5	3	2,5	2
Physical disturbance to seabed	1	2,8	4	4	4	4	4	1	4	1	3	3	3	1	3	3,3	4	0,03	3	4	1	3	3	3	3	0,6	0	3	1,5	1
Input of nutrients	3	4	3	4	3	4	3	1,5	3	1	3	2,5	3	1	3	3	4	2	2,5	2	1	2	2	2	2	3	1	1	1	0,6
Introductions of non-indigenous species	3	3	3,5	3	3	3	3	1	3	1	3	2,6	3	0	2,8	2	3	1	3	2	0	3	2	2	2	2	0	1	2	2
Changes to hydrological conditions	3	3	3	3	2	4	3	1	3	0,05	3	2	2	1	2	2	2,5	1	2	2	1	2	2	1	2	1,5	1	1	1	1
Disturbance of species due to human presence	2,1	2	2	1	2	3,5	2	3	2	3	2	1	2	3	2	1	2,5	4	1	1	3	1	1	0,5	1	0	2	0	0	0
Input of microbial pathogens	3	2,5	2	2	2	2	2	1,5	2	2	2	2	2	2	2	1	1	1,5	2	1	1	2	1	1	1	2	1,5	0	0	0
Bycatch by pelagic towed gears	4	3	1	1	0	0	1	4	1	3,9	0,5	0	1	3	0	0	0	3	0	0	3	0	0	0	0	3	2	1	1	0
Input of impulsive anthropogenic sound	3	2	1	1	1	0	0	3	0	5	0	0	0	5	0	0,9	0,5	3	0	0	4	0	0	0	0	2	5	0	0	0
Input of continuous anthropogenic sound	3	2	0	1	1	0	1	3	0,8	4	0,8	1	0,8	4	1	1	0	3	0	0	3	0	0	0	0	2	4	0	0	0

Table S6. Comparison of ecosystem sensitivity estimates among the marine regions. The table gives non-parametric 1-way ANOVA Savage and Siegel-Tukey test results (lower p-value of the two tests selected) for sensitivity scores among the regions. Regions not having a feature were omitted from the analysis.

	Circalittoral coarse sediment	Circalittoral mixed sediment	Circalittoral mud	Circalittoral rock and biogenic reef	Circalittoral sand	Infralittoral coarse sediment	Infralittoral mixed sediment	Infralittoral mud	Infralittoral rock and biogenic reef	Infralittoral sand	Offshore circalittoral coarse sediment	Offshore circalittoral mixed sediment	Offshore circalittoral mud	Offshore circalittoral rock and biogenic reef	Offshore circalittoral sand	Bathyal	Abyssal	Small toothed cetaceans	Deep diving toothed cetaceans	Baleen whales	Seals	Turtles	Breeding birds	Fish	Saltmarshes	Seagrasses	Cold-water corals and other coralligenous formations	Seamounts	Coastal water column habitat	Offshore water column habitat	*% less than 0.05*
Introductions of non-indigenous species	>0.1	>0.1	>0.1	>0.1	>0.1	<0.1	<0.1	>0.1	<0.05	>0.1	>0.1	>0.1	>0.1	>0.1	>0.1	>0.1	>0.1	>0.1	>0.1	>0.1	>0.1	>0.1	>0.1	<0.1	>0.1	>0.1	>0.1	>0.1	>0.1	>0.1	*3%*
Input of microbial pathogens	>0.1	>0.1	>0.1	>0.1	>0.1	<0.1	<0.1	>0.1	<0.1	<0.1	>0.1	>0.1	>0.1	>0.1	>0.1	>0.1	>0.1	>0.1	>0.1	>0.1	<0.1	>0.1	<0.05	<0.1	<0.05	<0.05	>0.1	>0.1	>0.1	<0.05	*13%*
Disturbance of species due to human presence	>0.1	>0.1	>0.1	>0.1	>0.1	<0.1	<0.1	>0.1	<0.05	<0.05	>0.1	>0.1	>0.1	>0.1	>0.1	<0.1	>0.1	>0.1	>0.1	>0.1	<0.05	<0.05	<0.1	>0.1	>0.1	>0.1	>0.1	>0.1	>0.1	<0.1	*13%*
Extraction of species by commercial fishing	<0.05	<0.1	<0.1	>0.1	>0.1	<0.05	<0.05	<0.1	>0.1	<0.05	<0.05	<0.1	>0.1	<0.05	<0.1	>0.1	>0.1	>0.1	>0.1	>0.1	<0.05	>0.1	>0.1	>0.1	<0.1	<0.05	>0.1	>0.1	>0.1	>0.1	*27%*
Bycatch by pelagic towed gears	>0.1	>0.1	>0.1	>0.1	<0.1	<0.05	<0.05	>0.1	>0.1	<0.1	>0.1	<0.1	<0.1	>0.1	>0.1	>0.1	>0.1	>0.1	>0.1	>0.1	>0.1	>0.1	>0.1	>0.1	<0.05	<0.1	>0.1	>0.1	>0.1	>0.1	*10%*
Bycatch by bottom touching mobile gears	>0.1	>0.1	>0.1	>0.1	>0.1	>0.1	>0.1	>0.1	>0.1	>0.1	>0.1	>0.1	>0.1	>0.1	>0.1	>0.1	>0.1	>0.1	>0.1	>0.1	>0.1	>0.1	>0.1	<0.1	>0.1	<0.1	<0.1	>0.1	>0.1	<0.1	*0%*
Physical loss of seabed	<0.05	<0.05	<0.1	<0.1	<0.1	<0.05	<0.05	<0.1	>0.1	<0.1	>0.1	>0.1	<0.1	>0.1	>0.1	>0.1	>0.1	>0.1	>0.1	>0.1	>0.1	>0.1	>0.1	>0.1	>0.1	>0.1	>0.1	>0.1	>0.1	>0.1	*13%*
Physical disturbance to seabed	<0.05	<0.05	<0.1	<0.05	<0.1	>0.1	>0.1	>0.1	<0.05	>0.1	<0.05	<0.05	>0.1	<0.05	<0.1	>0.1	>0.1	>0.1	>0.1	>0.1	<0.05	>0.1	<0.05	<0.05	>0.1	>0.1	>0.1	>0.1	<0.1	>0.1	*30%*
Changes to hydrological conditions	>0.1	>0.1	>0.1	>0.1	>0.1	<0.05	<0.05	<0.05	<0.05	>0.1	>0.1	>0.1	>0.1	>0.1	>0.1	>0.1	>0.1	>0.1	>0.1	>0.1	<0.1	>0.1	>0.1	<0.1	<0.1	>0.1	>0.1	<0.1	>0.1	>0.1	*13%*
Inputs of nutrients	>0.1	>0.1	>0.1	<0.05	>0.1	<0.05	<0.05	>0.1	>0.1	<0.1	>0.1	>0.1	>0.1	>0.1	>0.1	>0.1	>0.1	>0.1	>0.1	>0.1	<0.05	>0.1	>0.1	<0.1	>0.1	>0.1	>0.1	>0.1	>0.1	<0.1	*13%*
Input of hazardous substances	<0.05	<0.1	<0.05	<0.1	<0.1	>0.1	<0.05	<0.05	>0.1	<0.1	<0.1	<0.1	<0.1	<0.1	>0.1	>0.1	>0.1	>0.1	>0.1	>0.1	>0.1	>0.1	>0.1	<0.1	>0.1	<0.05	>0.1	>0.1	>0.1	<0.1	*13%*
Input of continuous anthropogenic sound	<0.1	<0.1	<0.05	>0.1	<0.05	>0.1	>0.1	>0.1	>0.1	>0.1	>0.1	>0.1	<0.1	<0.1	<0.1	>0.1	>0.1	>0.1	<0.05	>0.1	<0.1	>0.1	>0.1	>0.1	<0.05	<0.05	>0.1	>0.1	<0.05	<0.05	*23%*
Input of impulsive anthropogenic sound	>0.1	>0.1	>0.1	>0.1	>0.1	>0.1	>0.1	>0.1	>0.1	>0.1	>0.1	>0.1	>0.1	>0.1	>0.1	>0.1	>0.1	>0.1	>0.1	>0.1	>0.1	>0.1	>0.1	>0.1	<0.05	<0.05	>0.1	>0.1	<0.05	>0.1	*10%*
Sea surface temperature anomalies	>0.1	>0.1	>0.1	>0.1	>0.1	>0.1	>0.1	>0.1	>0.1	>0.1	>0.1	<0.05	>0.1	>0.1	>0.1	>0.1	>0.1	>0.1	>0.1	>0.1	>0.1	<0.1	<0.1	>0.1	>0.1	>0.1	>0.1	>0.1	>0.1	>0.1	*7%*
% less than 0.05	*29%*	*14%*	*14%*	*14%*	*7%*	*36%*	*43%*	*14%*	*36%*	*14%*	*14%*	*14%*	*0%*	*14%*	*0%*	*0%*	*0%*	*0%*	*7%*	*0%*	*29%*	*7%*	*14%*	*14%*	*29%*	*29%*	*0%*	*0%*	*14%*	*14%*	

Appendix S3. Comparison of the assessment products

Pressure and combined effect assessments have been made in European seas since the global assessment (Halpern et al. 2008). Stelzenmüller et al. (2015) showed how the data accuracy is a major source of uncertainty in CEAs. We assumed that the data accuracy depends on the scale of the assessment area, e.g. global assessments do not likely have access to as accurate data layers as regional ones. We compared outcomes of four assessments – the global (Halpern et al. 2015), the European (this study), the Baltic Sea (HELCOM 2018) and Danish (Andersen et al. 2020) – and evaluated whether the outcomes differ from each other in two ways: (1) comparing by a hot spot analysis in the ArcGIS software whether high and low effect areas were similarly identified in each CEA and (2) calculating actual differences in the CEA grid cells. Table S7 lists the characteristics of the four studies, figures S4 A-D present the hot spots identified by the analysis, and figure S5 presents the differences in effect values both as maps and in histogram format.

Table S7. Characteristics of the four combined effect assessments compared in this study.

CEA study	Number of pressure layers	Comment on pressure layers	Number of ecosystem layers	Comment on ecosystem layers
Global	17	One riverine pollution pressure, 16 pressures reflect major maritime sectors and climate change.	20	10 broad habitat types, 10 other habitats (e.g. seamounts, coral reefs, saltmarshes)
European	15	23 pressures integrated to 15 categories	30	6 species groups, 19 broad habitat types, 5 other habitats
Baltic Sea	18	39 specific pressure layers integrated to 18 categories	36	7 species, 5 species habitats, 5 biotopes, 10 broad habitat types, 9 other habitats
Danish	35	Pressures reflect human activities and were not integrated.	47	36 species/species groups, 9 broad habitat types, 2 other habitats

To prepare the CEA assessment products, we worked with raster layers. We first clipped the global assessment to the European assessment area and transformed the European one to the resolution of the global one (1 km × 1 km). To compare the European CEA with the Baltic CEA, we clipped the European assessment to the Baltic Sea assessment area and transformed it to 1 km × 1 km resolution. The Danish and the Baltic CEAs both were in 1 km × 1 km resolution and the Baltic one was clipped to the extent of the Danish one. We re-scaled all the four assessments to a similar scale between 0 and 1. We used the ArcGIS hot spot analysis tool with similar settings in each case to identify high and low effect areas in each of the maps, and then visually compared locations of the identified hot spots. Secondly, we compared CEA values in each grid cell by subtracting them by raster calculator and made new maps of the differences. Histograms of the difference scores further informed of the scale of the differences. The areas of highest differences were qualitatively evaluated, hence informing if any of the four assessments have given false management advice.

Figure S4. Hot spot analysis of the four CEAs: S3A for the global CEA, S4B for the European CEA, S4C for the Baltic CEA and S4D for the Danish CEA.

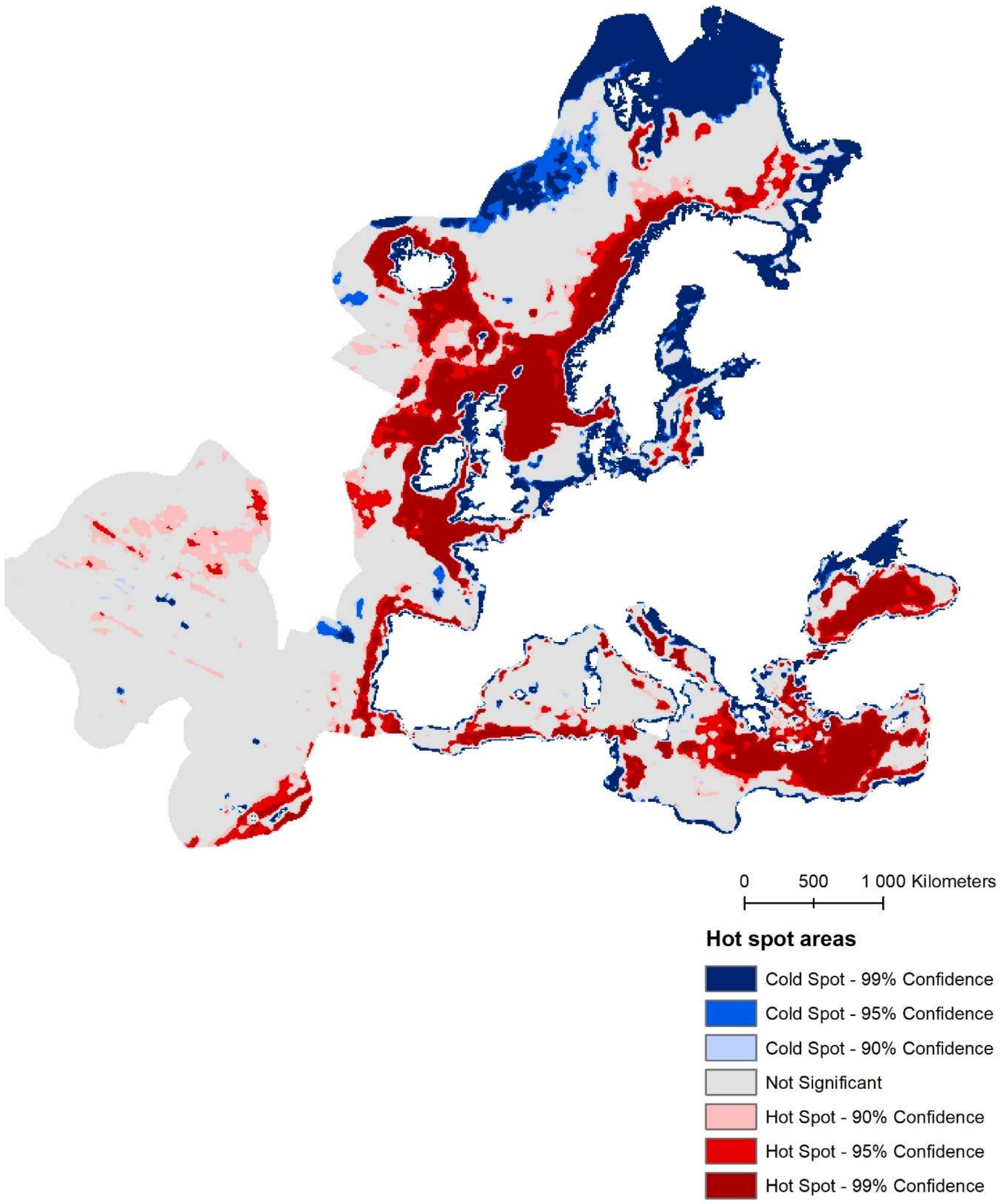

Figure S4 A. Hot spots in the global CEA which is limited to European marine area.

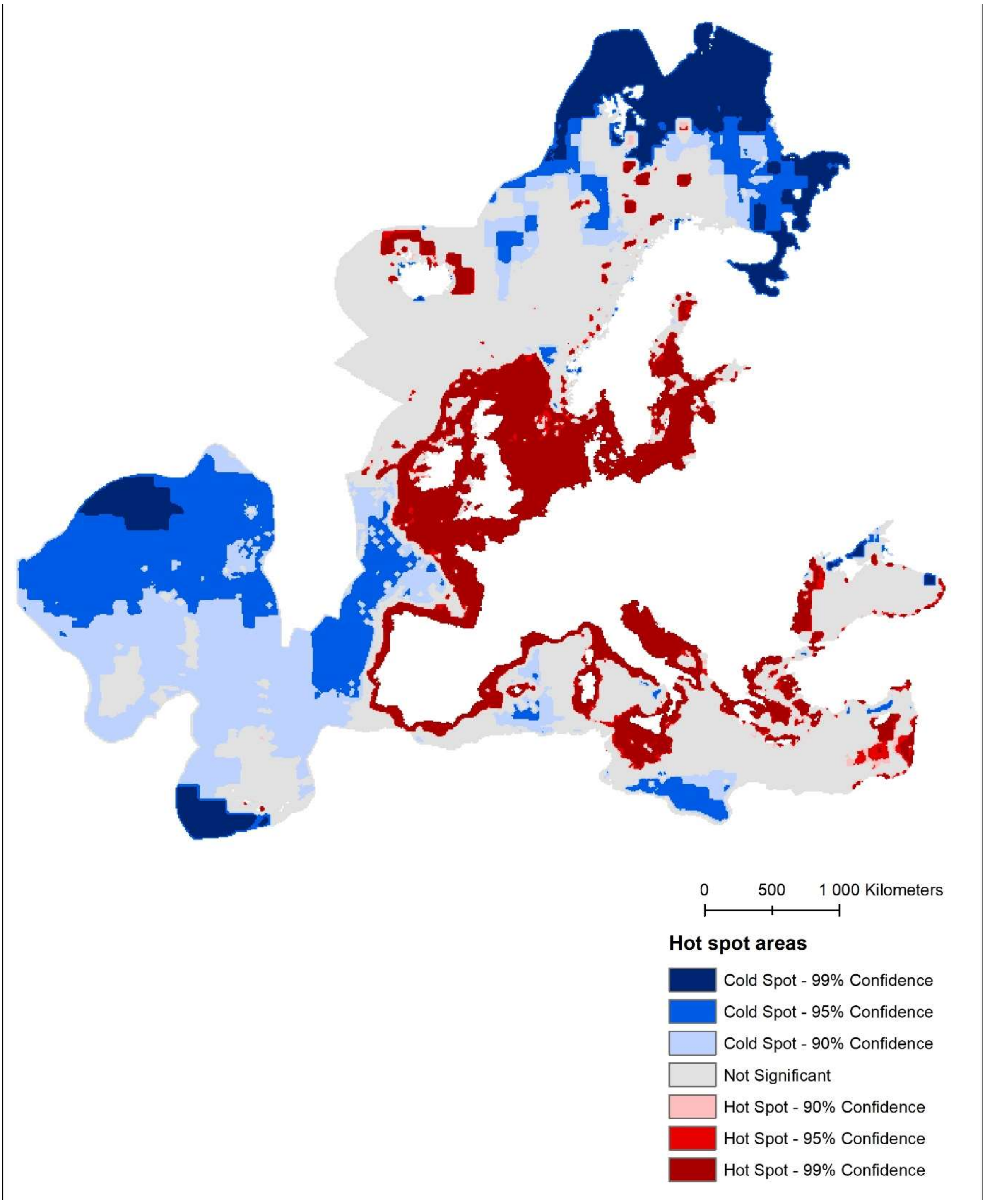

Figure S4 B. Hot spots in the European CEA.

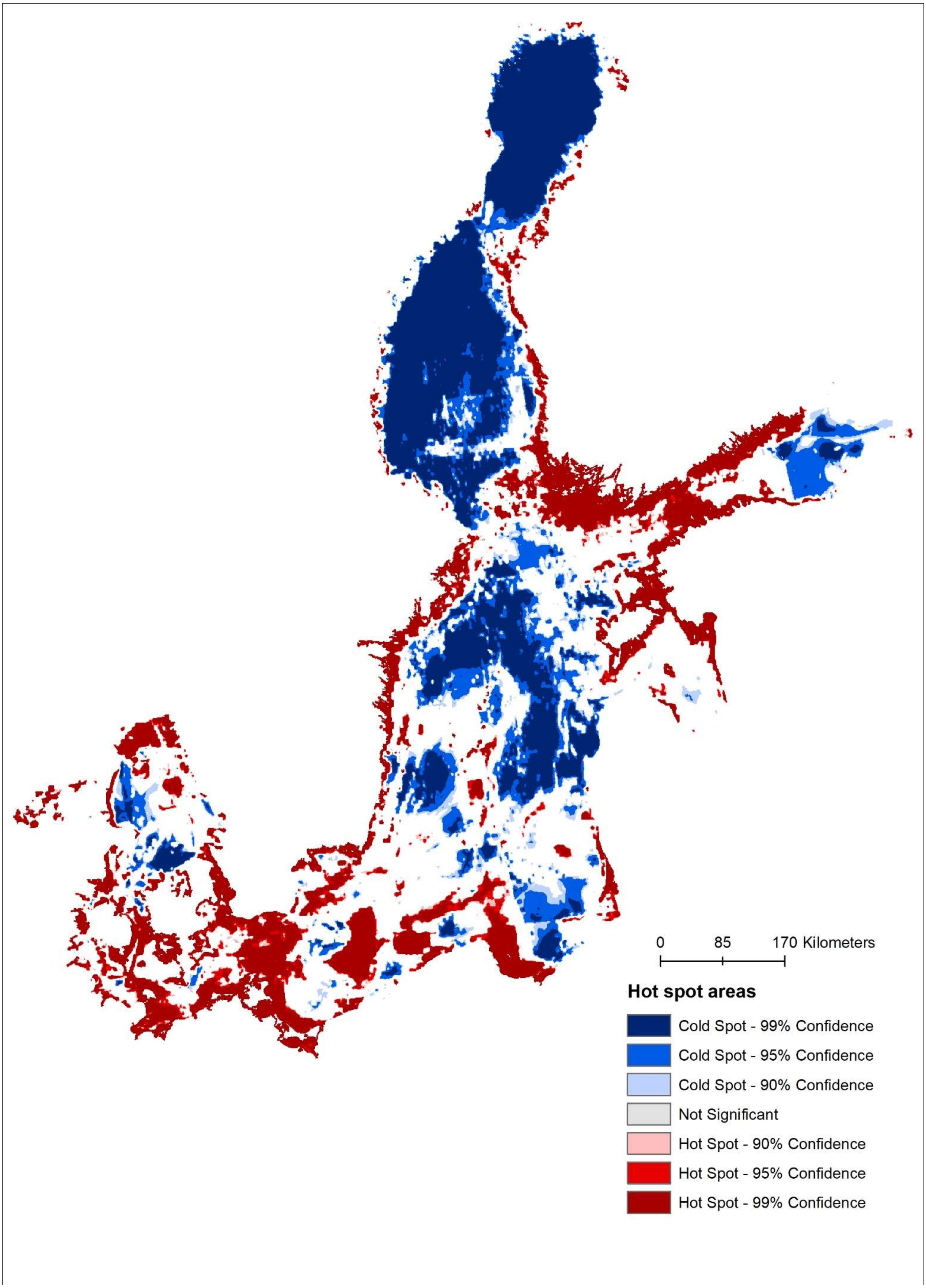

Figure S4 C. Hot spots in the Baltic Sea CEA.

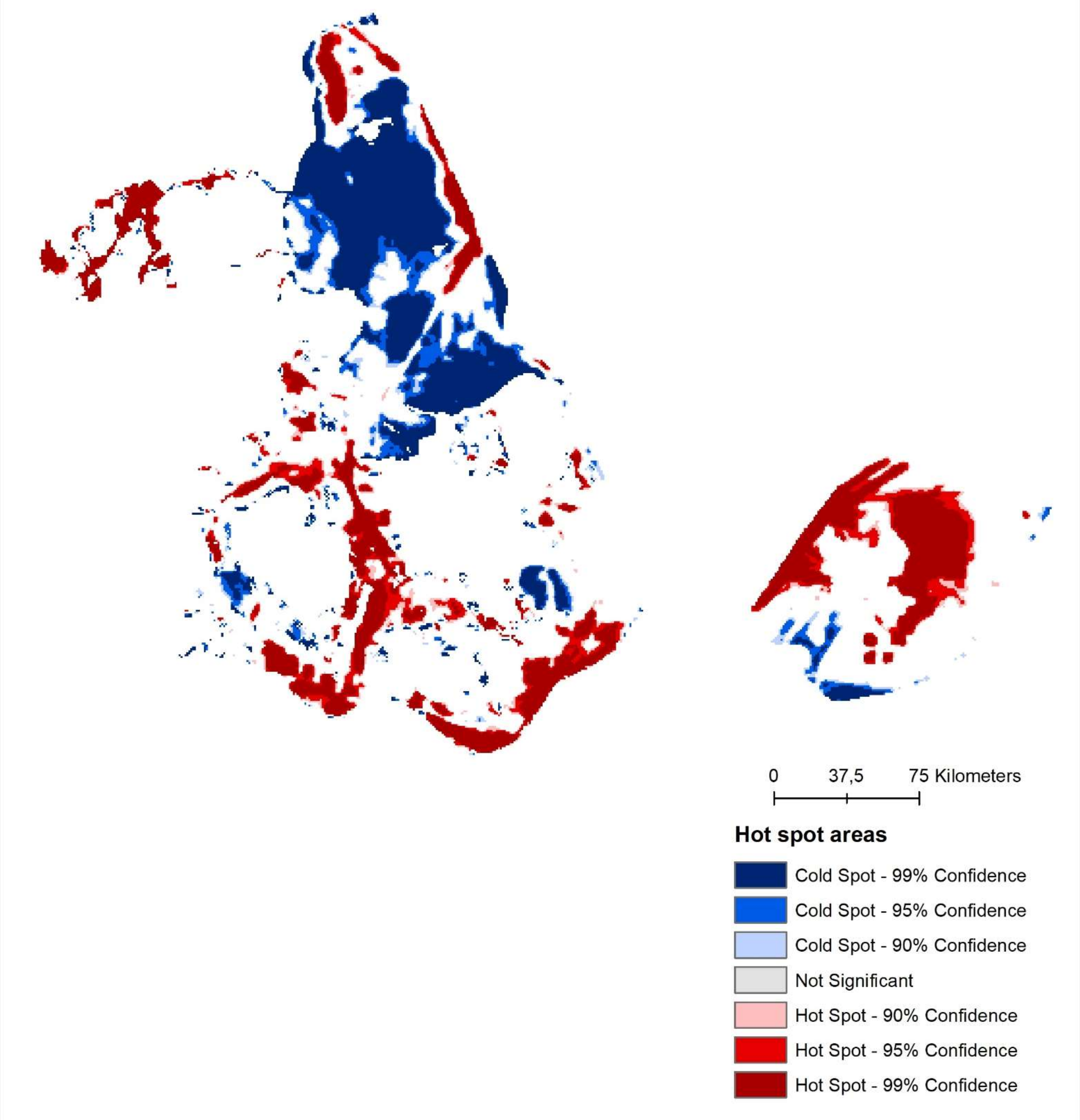

Figure S4 D. Hot spots in the Danish CEA.

The hotspot analysis showed that the global CEA differed most from the others. It missed the high-effect areas from the southern North Sea, Danish Straits and the northern shelf waters of the Mediterranean Sea (Figure S4 A). It also suggested high-effect areas to the Norwegian Sea while the European CEA did not consider that highly affected (Figure S4 B). Visual comparison of hotspots among the other three CEAs does not indicate big differences,

In pairwise comparisons, CEA values were subtracted from each other. Also this analysis showed that the European pressure assessment differed from the global one (Figure S5 A). The main difference was a major overestimation of the pressure effects by the global CEA in offshore areas but also the underestimation by the global CEA in shelf areas and coastal waters is clearly visible. As the offshore areas are wider, the differences were predominantly overestimations as seen in the histogram (54 % of the area differed by more than 25 %; Figure S5 D).

Also, the Baltic Sea assessment outcome showed differences against the European assessment (Figure S5 B). The European assessment typically overestimated, i.e. indicated higher effects, in offshore areas (28 % of area was overestimated by >25 % difference; Figure S5 E).

The Danish national assessment of combined effects had clearly higher effect estimates than the Baltic Sea CEA (Figure S5 C). This is seen in the histogram of difference scores which indicate high underestimation by the Baltic Sea CEA; 91% of Danish marine area differed by more than 25% higher scores in the Danish than Baltic assessment (Figure S5 F). The difference was not only coastal but also in the offshore areas.

The differences between the global CEA and the other CEAs was caused by the lack of data in the global model. This lack of data is obvious from the Halpern et al. (2008) data description and were visible in Europe in the southern North Sea and in the coastal areas. However, the vast overestimation of combined effects in the offshore waters (as shown in Figure S5 D) are probably also a result of limited amount data. As the global CEA was normalized to the same scale as the other CEAs, the limited range of values were expanded between 0 and 1 and wide offshore areas received as high scores as the real hot spots in the other CEAs. The same difference was visible also between the European and the Baltic CEA, as the Baltic coastal data was more accurate and thus the high scores more limited in space (Figure S5 E). This shows how difficult it is to compare to different indices if no common thresholds can be used to scale them properly.

The differences between the Danish and the Baltic Sea CEAs stem from another factor: the Danish CEA had many more offshore pressure layers which caused higher index scores in the offshore areas and thus higher index results compared to the Baltic assessment which had only a few offshore pressure layers (Figure S5 F). Such choices in input layers may greatly affect the CEAs (Stock & Micheli 2016) and make their detailed comparisons difficult. As Halpern and Fujita (2013) explain, the CEA method is sensitive to the choice of input layers.

The European marine CEA builds on a vast data set of pressures, habitats and species which has been collected under the European Union's marine knowledge 2020 programme (e.g. EMODnet data portals) and by the four regional sea conventions (Baltic Sea: https://helcom.fi/baltic-sea-trends/data-maps/, NE Atlantic: https://odims.ospar.org/, Mediterranean Sea: http://web.unep.org/unepmap/unepmap/resources/map-it-tools). From this starting point, it is not surprising that the European results are sharper in contrasts than the global CEA (Halpern et al. 2008, 2015). Similarly, the national data sources allow more data layers and finer scale assessments.

Figure S5. Cumulative effect assessments: (A) The CEA in the global scale clipped to Europe (Halpern et al. 2015), (B) the Baltic Sea assessment (HELCOM 2018a) and (C) the Danish assessment (Andersen et al. 2020). The European CEA is presented in Figure 1 of the main text. Pairwise comparisons as histograms of the difference: (D) Differences between the global and European CEA, (E) the European and Baltic CEA and (F) the Baltic and Danish CEA. Note that the long tails of the histograms up to ~±80% difference are not clearly visible. Positive difference is overestimation of combined effects by global vs. Europe (D), European vs. Baltic Sea (E) and Baltic vs. Denmark (F) and negative difference is the underestimation, respectively.

Panel A: Global CEA	Panel D: Difference in global and European CEAs
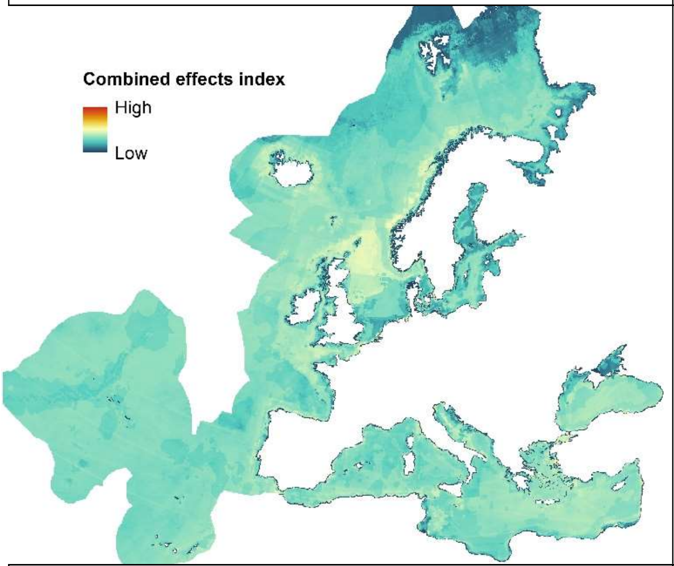	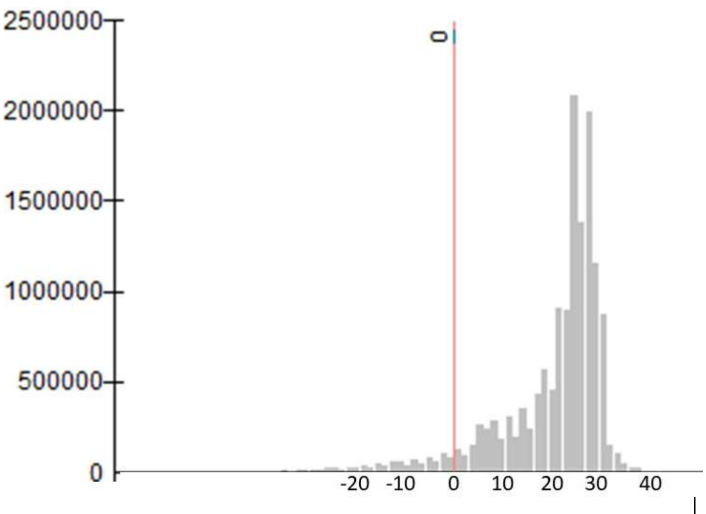
Panel B: Baltic Sea CEA	Panel E: Difference in European and Baltic CEAs

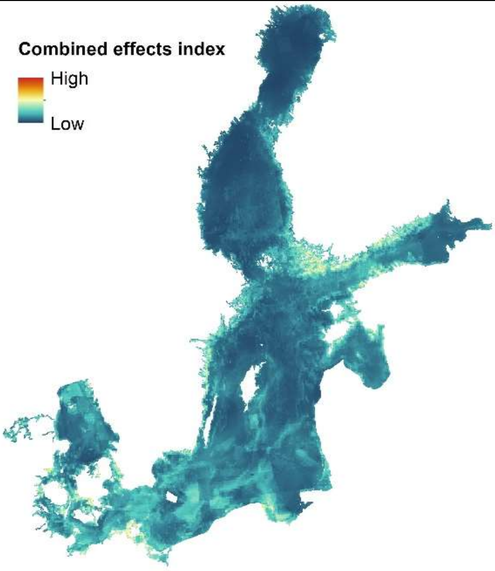

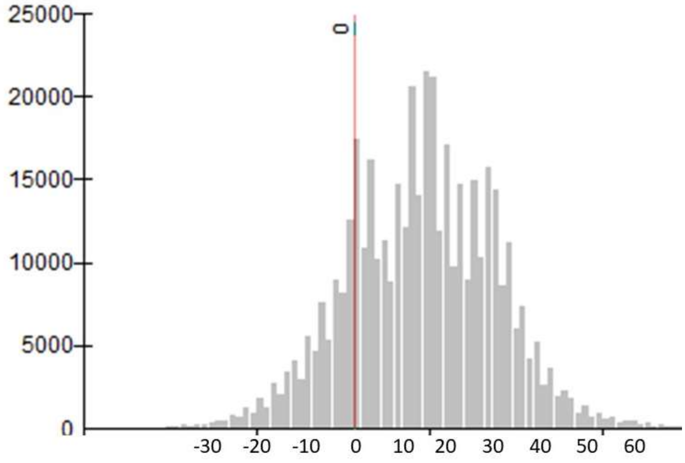

Panel C: Danish CEA

Panel F: Difference in Baltic and Danish CEAs

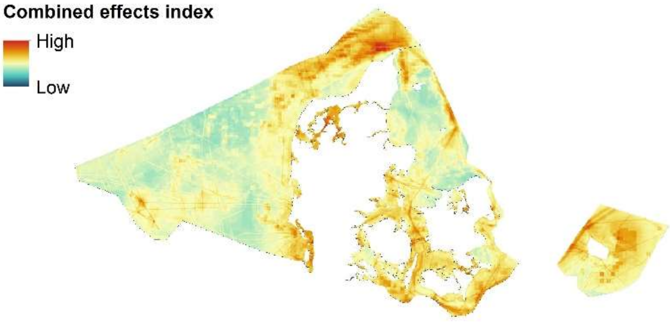

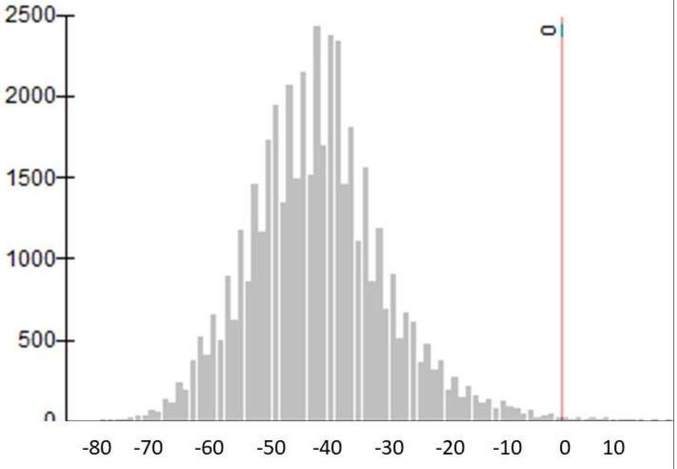

References:

Andersen, J.H., Al-Hamdani, Z., Harvey, E.T., Kallenbach, E., Murray, C. & Stock, A. (2020): Relative impacts of multiple human stressors in estuaries and coastal waters in the North Sea–Baltic Sea transition zone. Science of the Total Environment 704, 135316.

Borysova, O., Kondakov, A., Paleari, S., Rautalahti-Miettinen, E., Stolberg, F. &Daler, D. (2005): Eutrophication in the Black Sea region; Impact assessment and Causal chain analysis. Global International Water Assessment. University of Kalmar, Kalmar, Sweden.

HELCOM (2018): Thematic assessment of cumulative impacts on the Baltic Sea 2011-2016. Baltic Sea Environment Proceedings No. 159. Available at: http://www.helcom.fi/baltic-sea-trends/holistic-assessments/ state-of-the-baltic-sea-2018/reports-and-materials/

Halpern, B. S., Frazier, M., Potapenko, J., Casey, K. S., Koenig, K., Longo, C., et al. (2015): Spatial and temporal changes in cumulative human impacts on the world's ocean. Nat. Commun. 6, 7615.

Halpern, B. S. & Fujita, R. (2013): Assumptions, challenges, and future directions in cumulative impact analysis. Ecosphere 4. doi: 10.1890/ES13-00181.

Halpern, B.S., Walbridge, S., Selkoe, K.A., Kappel, C.V., Micheli, F., D'Agrosa, C., et al. (2008): A global map of human impact on marine ecosystems. Science 319, 948–952.

Kaplan, A., Kushnir, Y., Cane, M. & Blumenthal, M. (1997): Reduced space optimal analysis for historical data sets: 136 years of Atlantic sea surface temperatures. J. Geophys. Res. 102 835-860.

Katsanevakis,S.,Wallentinus, I., Zenetos, A., Leppäkoski, E., Çinar, M.E., Oztürk, B., Grabowski, M, Golani D, & Cardoso, A.C. (2014): Impacts of invasive alien marine species on ecosystem services and biodiversity: a pan European review. Aquatic Invasions 9: 391–423.

Mena, C., Reglero, P., Hidalgo, M:, Sintes, E., Santiago, R., Martin, M., Moyá, G. & Balbín, R. (2019): Phytoplankton Community Structure Is Driven by Stratification in the Oligotrophic Mediterranean Sea. Front. Microbiol., 24 July 2019 | https://doi.org/10.3389/fmicb.2019.01698

Stelzenmüller, V., Vega Fernández, T., Cronin, K., Röckmann, C., Pantazi, M., Vanaverbeke, J., et al. (2015): Assessing uncertainty associated with the monitoring and evaluation of spatially managed areas. Marine Policy 51: 151-162.

Stock, A. & Micheli, F. (2016): Effects of model assumptions and data quality on spatial cumulative human impact assessments. Global Ecology and Biogeography 25: 1321-1332.

Strobl, R.O:, Somma, F., Evans, B.M. & Zaldívar, J.M. (2009): Fluxes of water and nutrients from river runoff to the Mediterranean Sea using GIS and a watershed model. Journal of Geophysical Research 114, G03012.
